# A europium metal–organic framework for dual Fe^3+^ ion and pH sensing

**DOI:** 10.1038/s41598-022-15663-z

**Published:** 2022-07-14

**Authors:** Linda Rozenberga, William Skinner, David G. Lancaster, Witold M. Bloch, Anton Blencowe, M. Krasowska, David A. Beattie

**Affiliations:** 1 Future Industries Institute, University of South Australia, Mawson Lakes, SA 5095 Australia; 2 Department of Chemistry and Physics, University of Adelaide, Adelaide, SA 5000 Australia; 3 Applied Chemistry and Translational Biomaterials Group, UniSA Clinical and Health Science, University of South Australia, Adelaide, SA 5000 Australia

**Keywords:** Analytical chemistry, Materials chemistry

## Abstract

Metal–organic frameworks (MOFs) with ratiometric sensing properties are desirable for many applications due to their intrinsic self-calibration. We report the re-assessment of the sensing properties of a MOF, originally reported as containing europium(III) and 2-hydroxyterephtalic acid, and having fluorescent ratiometric iron(III) sensing properties. Synchrotron single-crystal X-ray diffraction and proton nuclear magnetic resonance (^1^H NMR) spectroscopy revealed that the MOF is composed of 2-methoxyterephthalate, not 2-hydroxyterephthalate as originally reported. We found that the MOF exhibits a sensor turn-off response towards Fe^3+^ ion concentrations in the range 0.5–3.7 ppm (band 425 nm), and a turn-on response towards a decrease of pH from 5.4 to 3.0 (band 375 nm), both resulting from the addition of acidic Fe^3+^ salt solution to a MOF suspension. Thus, the ratiometric sensing properties and the originally proposed mechanism no longer apply; our work reveals a dynamic quenching mechanism for the fluorescence turn-off response due to the presence of Fe^3+^ ions, and a ligand protonation mechanism for the turn-on response to a decrease in pH. Our work highlights the importance of a thorough investigation of the structure of any newly synthesized MOF, and, in the case of potential sensors, their selectivity and any environmental effects on their sensing behavior.

## Introduction

The ability to selectively and precisely detect metal ions is crucial to many applications relating to biomedicine^1^, nuclear science^2^, and mineral processing^3^. However, the existing analytical methods are expensive, laborious, and time-consuming, particularly in the case of ferric ion concentration measurements^4^. Fe^3+^ ion detection is vital in environmental and biomedical analysis, as excess iron contents have been linked to the development of diseases such as Parkinson’s syndrome^5^, Alzheimer’s disease^1,5^ and cancer^6^. In the mineral processing industry, regulating Fe^3+^ ion concentrations can be used to optimize mineral recovery rates, specifically in copper^7^, uranium^8^ and zinc^9^ leaching processes. In this regard, fluorescent sensors for metal ions have received significant interest due to their ability to provide a simple, sensitive, selective, precise and economical method for real-time measurement of very low ion concentrations without any pre-treatment of the sample^2^.

Metal–organic frameworks (MOFs), also called porous coordination polymers, are a class of crystalline materials composed of metal ions or clusters interconnected by organic ligands. Their crystalline, porous structure and isoreticular synthesis make them excellent candidates for a wide range of applications including gas storage and separation^10,11^, catalysis^12,13^, biomedicine^14,15^ and chemical sensing^16–18^. Lanthanide ion containing MOFs, in particular, offer unique fluorescent properties for metal ion detection, including long lifetimes, characteristic sharp emissions, large Stokes shifts, and high color purity with high quantum yields in the near-infrared and visible regions^19^.

Generally, the sensing of metal ions by lanthanide MOFs occurs by interaction of the analyte with the open metal sites or ligand backbone. In the case of the latter, metal ions commonly interact with Lewis basic sites of the ligand, such as NH_2_, pyridine or OH moieties^16,20,21^. This can produce fluorescence enhancement (‘‘turn-on”), fluorescence quenching (‘‘turn-off”) or ratiometric fluorescence detection (accurate detection through self-calibration with two emissive bands). Ratiometric sensors are the most desirable types of fluorescence sensors since the detection of the target analyte is not affected by the sensors’ concentration, the environmental conditions and divergence in optical components between instruments, factors that can negatively affect both ‘‘turn-on” and ‘‘turn-off” sensing^2,22^.

Given their stability, porosity and promising sensing properties, it is vital for the sensing behavior of fluorescent MOFs to be reproducible and fully understood. Our interest in preparing composite ratiometric sensors for Fe^3+^ ion detection in devices deployable in mineral processing led us to a Eu MOF (EuOHBDC) reported by Xu et al*.*^23^, the first MOF reported in the literature as a ratiometric ferric ion sensor (for a summary of recent reported MOFs for ferric ion sensing, refer to the Supporting Information (SI, Table S1). This MOF, which was synthesized from 2-methoxyterephthalic acid and Eu(NO_3_)_3_, was described to possess a rare ligand-based ratiometric response to Fe^3+^ ion concentration, resulting from interactions between Fe^3+^ ions and the OH group of the ligand, whereby the OH group was reported to arise from an in-situ demethylation of the methoxy moiety during MOF synthesis.

Herein, we present a reassessment of the structure and sensing mechanism of ‘EuOHBDC’*.* Our analysis of this Eu MOF revealed that the previously reported chemical structure is incorrect; synchrotron single-crystal X-ray diffraction coupled with NMR spectroscopy unequivocally revealed that the in-situ demethylation of 2-methoxyterephthalic acid does not occur, and the methoxy moiety of the ligand is preserved in the MOF structure. Although the three-dimensional (3-D) network of the MOF is unchanged, the absence of OH metal binding sites led us to revise the properties and sensing mechanism that were attributed to this material. We discovered that EuOHBDC (herein re-named EuBDC-OMe) is not a ratiometric sensor for Fe^3+^ ions, as originally reported. Instead, the MOF possesses “turn-on” pH sensing and “turn-off” Fe^3+^ ion sensing capabilities.

## Experimental

### Materials

All chemicals were used as received unless otherwise stated. Ultrapure water (Milli-Q, resistivity (ρ) = 18.2 MΩ‧cm @ 25 °C) was obtained from Milipore Advantage A10. Methanol (HPLC grade) was purchased from UNICHROM Ajax 2314. Dimethyl sulfoxide—D6 (D, 99.9%) was obtained from Cambridge Isotope Laboratories. Deuterium chloride solution (35 wt% in D_2_O, ≥ 99% atom % D), KMnO_4_ (≥ 99%), 2,5-dimethylanisole (99.0%), and Eu(NO_3_)_3_·6H_2_O (99.9%), FeCl_2_ (99.99%) and FeCl_3_ (99.99%) were purchased from Sigma Aldrich (Australia). HCl (36.5%) was purchased from ACI Labscan. KCl (99.0%), NaCl (99.7%), CaCl_2_·2H_2_O (98.0%), ZnCl_2_ (98.0%), CuCl_2_·2H_2_O (97.0%), AgNO_3_ (99.0%) and MnSO_4_·H_2_O (98.0%) were purchased from ChemSupply (Australia). MgCl_2_·6H_2_O (99.0%), Pb(NO_3_)_2_ (99.0%) was purchased from Fisher Scientific. For synthesis of EuBDC-OMe, Eu(NO_3_)_3_ and 2-methoxyterephthalic acid were used. 2-Methoxyterephthalic acid was prepared according to a literature procedure^23^ using 2,5-dimethylanisole and KMnO_4._

### Synthesis of EuBDC-OMe

The synthesis of EuBDC-OMe was carried out according to the original report by Xu et al*.*^23^ with modifications to the synthesis temperature and concentration (SI, Fig. S1)*.* In our process, the optimized conditions afforded the MOF in 38% yield as compared to 4% using the original conditions. Briefly, Eu(NO_3_)_3_·6H_2_O (900 mg, 2.67 mmol) and 2-methoxyterephthalic acid (300 mg, 1.53 mmol) were suspended in ultrapure water (150 mL) in a Teflon-lined autoclave reactor (200 mL), which was sealed and heated at 160 °C for 3 days. After slow cooling to the room temperature, the pink platelet-shaped crystals were washed with water (3 × 2 mL), followed by methanol (3 × 2 mL) to remove unreacted reagents, then dried under high vacuum to afford EuBDC-OMe, 166 ± 45 mg (38.5%; n = 5). Synthesis using 2-hydroxyterephthalic acid under the same conditions did not yield MOF crystals.

### Physical measurements

NMR spectra were recorded using an Agilent 500 MHz NMR spectrometer (^1^H at 500 MHz). Residual solvent peaks were used as an internal reference for ^1^H NMR spectra (DMSO δ_H_ 2.50 ppm).

Attenuated Total Reflection Fourier transform infrared spectroscopy (ATR FTIR) spectra were obtained using a DuraSamplIR 3 mm diameter diamond-faced 3 reflection ZnSe internal reflection element (IRE) (ASI sensIR Technologies), mounted in a Varian 670-IR FTIR spectrometer. EuBDC-OMe suspensions in water and HCl solutions were prepared by placing EuBDC-OMe crystals in pH adjusted solutions (EuBDC-OMe 0.01% w/v) and soaking for 24 h. The suspensions (without solvent evaporation) were deposited onto the clean diamond surface for analysis. Identical solvent spectra to the ones used for the preparation of suspensions were obtained prior to suspension measurements and manually subtracted during data processing. UV–Visible spectra were recorded using a Thermo Evolution 201 UV–Vis Spectrophotometer.

Thermogravimetric analysis (TGA) was carried out using a Discovery Thermogravimetric Analyzer (TA Instruments) at a heating rate of 10 K/min from 25 to 900 °C under a nitrogen atmosphere (BOC). The EuBDC-OMe particle morphology and size was visualized using a Zeiss Merlin FEG scanning electron microscope in analytical column mode and an electron high tension target 2 kV.

Powder X-ray diffraction (PXRD) data were collected on a Bruker D4 Endeavour powder diffractometer (Co-target, plate stage). Simulated powder X-ray diffraction patterns were generated from the single crystal data using Mercury 3.10. Gas sorption isotherm measurements were performed on a Micromeritics 3Flex Surface Characterization Analyzer. UHP Nitrogen (99.999%, BOC) was used for all measurements. Temperatures were maintained at 77K using a cryo-cooler. The isotherms were then analyzed to determine the Brunauer Emmett-Teller (BET) surface area and pore-size distribution using the MicroActive software (Version 3.00, Micromeritics Instrument Corp. 2013).

X-ray photoelectron spectroscopy (XPS) data were collected from EuBDC-OMe suspensions in ultrapure water (1 mg of MOF per 1 mL of H_2_O) and EuBDC-OMe suspensions in Fe^3+^ ion containing solutions (1 mg of MOF per 1 mL of 4 mM FeCl_3_·6H_2_O, MOF soaked for 72 h prior to analysis). The suspensions were deposited by placing a droplet (~ 0.5 mL) of the sample on the sample stubs and allowing it to air-dry. XPS was conducted using a Kratos Axis-Ultra spectrometer employing a monochromatic Al Kα X-ray source (1486.6 eV) operated at 130 W. All samples were cooled, in vacuum, using a liquid-nitrogen-fed cold stage (temperature <  − 100 °C) to minimise photoreduction during exposure to X-rays during analysis^24^. Survey spectra were acquired with a pass energy of 160 eV and the atomic % of detected elements were quantified using the appropriate elemental sensitivity factors. High-resolution spectra were acquired with a pass energy of 40 eV. Charge neutralisation was employed throughout all analyses. Both surface concentration calculations and spectral fitting was accomplished using the CasaXPS software package (http://www.casaxps.com/).

### Single-crystal X-ray diffraction

Single crystals were mounted in paratone-N oil on a plastic loop. X-ray diffraction data for EuBDC-OMe were collected at 100(2) K on the MX-2 beamline of the Australian Synchrotron. Experimental details, materials, methods and X-ray experimental data can be found in SI, X-ray crystallography section.

### Luminescence studies

Luminescence excitation and emission spectra were obtained using a Shimadzu RF-6000 Spectrofluorophotometer, with spectral bandwidth for both excitation and emission set to 5 nm, and the photomultiplier tube (PMT) voltage set to high. For each luminescence measurement, the analyte was excited at a wavelength of 320 nm and the fluorescence emission spectrum was measured in the range of 335–800 nm. All luminescence and ion sensing experiments were performed on EuBDC-OMe suspensions in ultrapure water (0.01% w/v) that had been sonicated using a Soniclean sonicator bath (43 ± 2 kHz sweep bandwidth with 20 Hz pulses) at 25–35 °C for 90 min. Suspension volumes of 3 mL in quartz cuvettes were used for all measurements. For luminescence titration measurements, cation selectivity and the study of sensing changes versus temperature, a Shimadzu RF-6000 Constant-Temperature Single Cell Holder with stirrer was used. Cation solutions (0–75 µM) were incrementally added to the 3 mL aqueous suspensions of EuBDC-OMe under constant agitation.

The lifetime measurements of EuBDC-OMe were performed with and without Fe^3+^ ions present, and at various pH levels (decreased by the addition of HCl). Fluorescence lifetimes were measured at 430 nm using a monochromator, amplified silicon detector, and oscilloscope. The experimental details, materials and analysis methods for measurement of the fluorescence lifetimes can be found in SI, Time domain fluorescence lifetime measurement method section.

## Results

### MOF synthesis, characterization and structure determination

In their paper, Xu et al. reported that a MOF composed of Eu^3+^ and 2-hydroxyterephthalate is synthesized from Eu(NO_3_)_3_ and 2-methoxyterephtalic acid^23^. MOF formation was proposed to be driven by an in-situ demethylation of the ligand’s methoxy group to produce an extended 3-D structure with the formula Eu_2_(BDC-OH) (BDC-OH = 2-hydroxyterephtalate). Following their original protocol—which involves heating the metal salt and ligand at 140 °C in water—we obtained the MOF as a crystalline solid with good correlation to the theoretical structure by PXRD (SI, Fig. S2).

**Figure 1:**
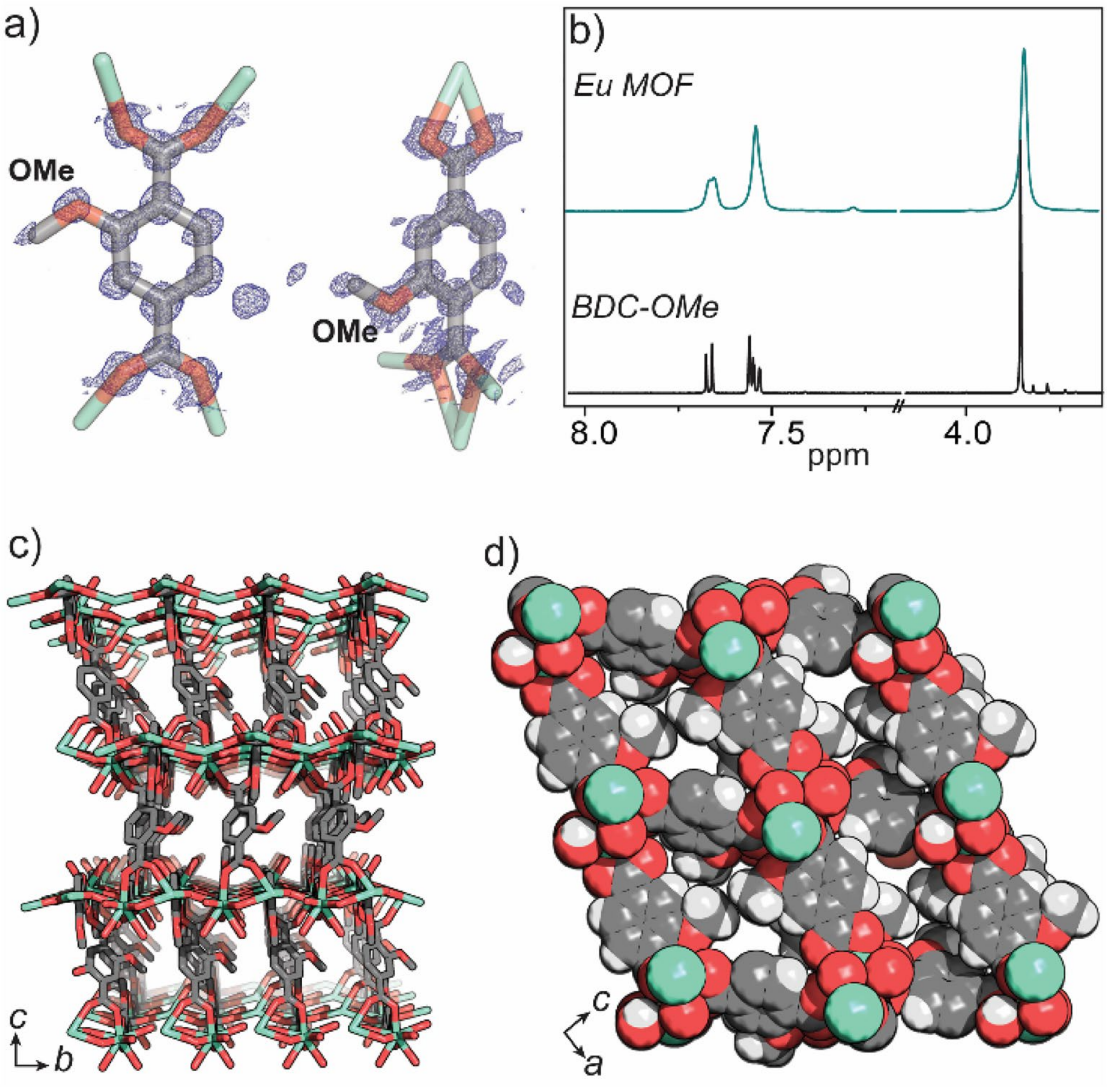
(**a**) an overlay of the F_obs_ map (blue) and BDC-OMe ligand as present in EuBDC-OMe; (**b**) ^1^H NMR spectra (500 MHz, DMSO-d_6_/4% DCl) showing the BDC-OMe ligand (bottom) and the digested EuBDC-OMe MOF (top); (**c**) synchrotron X-ray skeletal structure of EuBDC-OMe viewed along the c axis and (**d**) space-filling structure viewed along the b axis.

When we characterized this material by synchrotron single-crystal X-ray diffraction (SCXRD), we discovered an important discrepancy with the reported chemical structure of the MOF (SI, Table S2, Fig. S3). SCXRD analysis was performed on a desolvated MOF crystal, which improved the refinement of the ligand’s disorder as compared to the solvated crystals. The desolvated MOF had nearly identical unit cell parameters (*P*2_1_/*n*, *a* = 11.5, *b* = 6.8, *c* = 20.9, β = 102.8°) as compared to the previously reported data (*a* = 11.5, *b* = 6.8, *c* = 21.1, β = 103.3°)^23^, albeit with a slight contraction of the *c* axis^25^. Xu et al*.* reported a structure based on 2-hydroxyterephtalate, analysis of the data that we collected reveals that the MOF is composed of 2-methoxyterephtalate; electron density corresponding to the disordered methoxy groups can be clearly observed in the F_obs_ map (Fig. 1a, SI Fig. S4). The relatively low electron density associated with these methoxy groups relates to the rotational and positional disorder of the two crystallographically unique 2-methoxyterephthalate ligands. The other features of the structure are identical to the original MOF structure; each Eu^3+^ metal centre is coordinated by nine oxygen donors; eight from six 2-methoxyterephtalate ligands (two of these chelate) and one from a coordinated water molecule. Further evidence supporting the methoxy substituents of the ligand was obtained by ^1^H NMR spectroscopy. The ^1^H NMR spectrum of the acid digested MOF sample (in DMSO-d_6_/DCl) displayed resonances consistent with 2-methoxyterephthalic acid, as evidenced by the methoxy proton resonance at δ_H_ 3.83 ppm (Fig. 1b). A similar ^1^H NMR spectrum (of 2-methoxyterephthalic acid) was obtained by digesting a MOF sample synthesized at an elevated temperature of 160 °C (Fig. S5), once again revealing that the demethylation of the ligand (as reported by Xu et al.^23^) does not occur under the conditions for MOF synthesis. Combined, the synchrotron SCXRD data and ^1^H NMR experiments clearly show that this MOF is composed of 2-methoxyterephlatate and not 2-hydroxyterephlatate.

In our revised structure, the previously reported pores of 8.0 Å in diameter are now largely occupied by disordered methoxy substituents. These methoxy substituents, which point directly into the rhombus shaped pores of the MOF (along the *b* axis), reduce the pore dimensions to approximately 3.2 × 2.2 Å (Fig. 1c,d). To examine the porosity of EuBDC-OMe, we prepared a dehydrated sample by activation from MeOH at 100 °C. As shown by PXRD, the EuBDC-OMe MOF shows excellent stability transitioning from a hydrated state (post synthesis) to a dehydrated activated state (SI, Fig. S2). The 77K N_2_ adsorption profile of EuBDC-OMe is consistent with a typical type I isotherm with a BET surface area of 133 ± 2 m^2^/g (Figs. S6–S8). This modest value is consistent with the largely occupied pore space afforded by the disordered methoxy substituents.

Next, we assessed the pH stability of EuBDC-OMe; a practical Fe^3+^ ion sensing platform requires operation below pH 4.5 (ferric ions precipitate as Fe(OH)_3_ at pH values of > 4.5)^26,27^. PXRD revealed that the MOF is stable between pH 5.4 and 2.8 (SI, Fig. S9a), with degradation occurring below pH 2.8, as evidenced by new 2θ diffraction peaks at 7.4 and 15.0°, which corresponded to the crystalline salt of 2-methoxyterephtalic acid (confirmed by ^1^H NMR spectroscopy, SI, Fig. S9b). Decomposition of the MOF at this pH was corroborated by ATR FTIR which revealed the appearance of a vibration at 1690 cm^−1^ at pH < 2.8 associated with the COOH moiety of the ligand (SI, Fig. S10, Table S3). In the context of Fe^3+^ ion sensing, the chemical stability of EuBDC-OMe is comparable to other fluorescent MOFs sensors, where decomposition is typically observed below pH 3–4^28–31^. TGA decomposition curve and observations can be found in SI (Fig. S11).

### Photoluminescence properties

**Figure 2:**
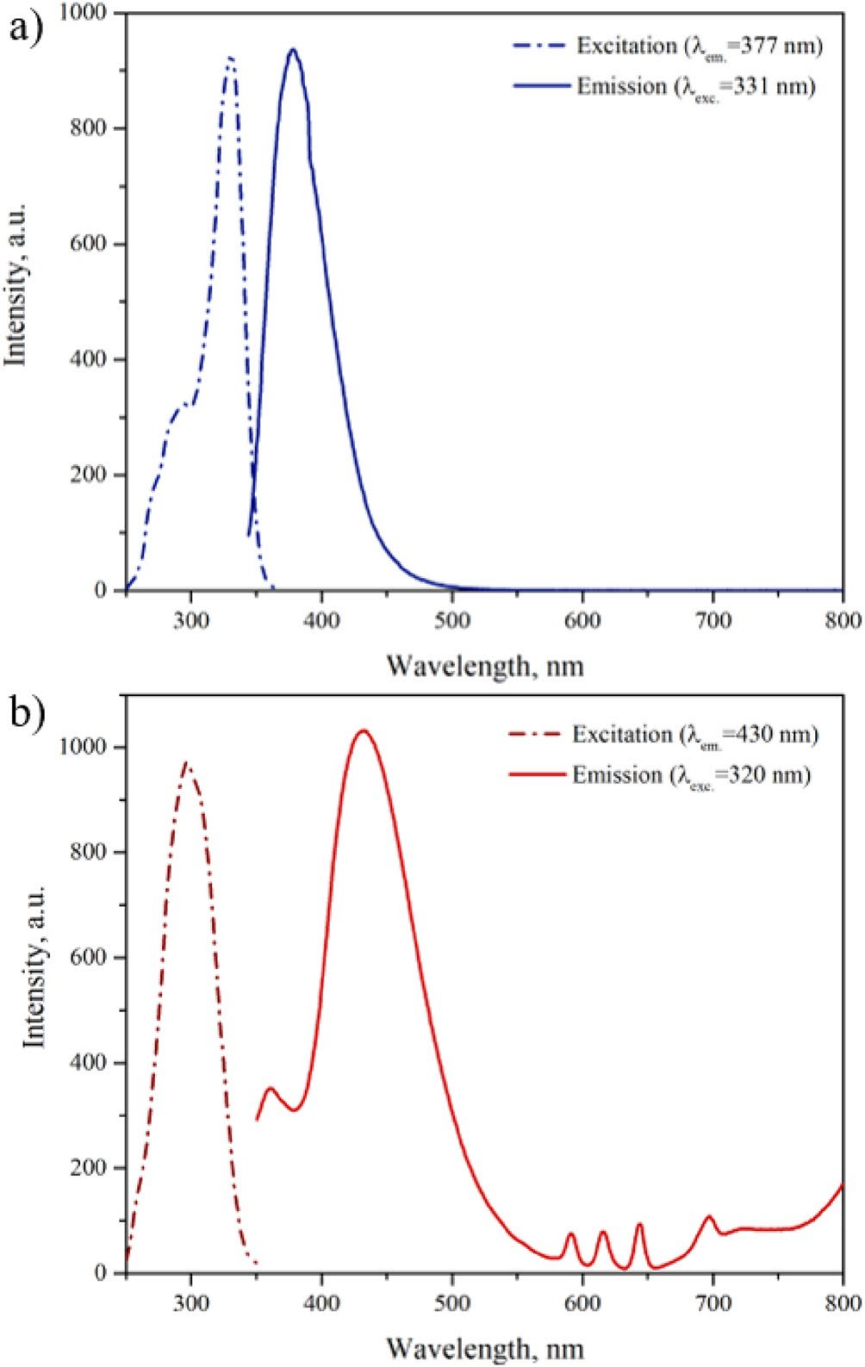
Excitation and emission spectra for (**a**) 2-methoxyterephthalic acid in water (0.01% w/v) and (**b**) the EuBDC-OMe MOF suspension in water (0.01% w/v).

Detailed assignment of the emission spectrum is essential for clarifying the sensor properties and detection mechanism. Our revised structure of the EuBDC-OMe MOF suggests that the mechanism behind its fluorescence quenching properties may be different compared to the original report.^23^ Therefore, we carefully investigated the luminescence properties of EuBDC-OMe as a particle dispersion in water (0.01% w/v at 21 ± 1 °C). The fluorescence excitation and emission spectra of EuBDC-OMe are identical to the ones originally reported by Xu et al.^23^ The emission spectra of 2-methoxyterephtalic acid in water (0.01% w/v) displays a strong fluorescence emission peak at 375 nm (λ_exc_ = 330 nm) (Fig. 2a), attributable to a π → π* transition^17,32^. When incorporated in the EuBDC-OMe MOF (Fig. 2b), the excitation maximum shifts to 320 nm. The emission spectra contains a weak ligand band at 375 nm and an intense, wide emission band at 430 nm (originally assigned to 2-hydroxyterephtalic acid)^23^ along with weak Eu^3+^ bands at 590, 615, 640 and 695 nm corresponding to ^5^D_0_ → ^7^F_1_, ^5^D_0_ → ^7^F_2_, ^5^D_0_ → ^7^F_3_ and ^5^D_0_ → ^7^F_4_ transitions, respectively^33^. The band at 640 nm overlaps with the second order grating Rayleigh scattering peak of water, an instrumental artefact from the diffraction grating of the monochromator^34^.

The broad, intense band at 430 nm was originally assigned to 2-hydroxyterephtalic acid emission. However, as discussed earlier, the synthesized MOF is composed of 2-methoxyterephtalate ligands, and this band is not present in the emission spectrum of 2-methoxyterephtalic acid. We assigned the 430 nm band to a ligand to metal charge-transfer (LMCT) state, based on the following considerations. In EuBDC-OMe, the LMCT state competes with linker-based luminescence, resulting in two emission bands. This phenomenon is common for MOFs with BDC-based ligands and the exact position of the charge-transfer bands depends on the nature of the ligand and the structure of the MOF^17,18,32,35,36^. The emission spectra of EuBDC-OMe indicates that the ligand sensitized lanthanide emission mechanism is not fully realized (Fig. 2b); the ligand fluorescence emission band at 375 nm indicates that the energy transfer to the Eu^3+^ is not complete^33^.

In the sensitization process of EuBDC-OMe, it would be expected that the excitation energy is absorbed by the 2-methoxyterephtalate ligand, and then shifted to the long-lived triplet state (T_1_) of the ligand via intersystem crossing. The appropriate excited levels of Eu^3+^ would then be populated by the energy derived from T_1_ to complete the photon conversion and the Eu^3+^-related radiative emission processes^37,38^. The presence of the LMCT emission band in EuBDC-OMe indicates that the energy difference between the ligand’s triplet state and the excited lanthanide state is not well matched, resulting in depopulation of the Eu^3+^ excited states, partial energy back transfer away from Eu^3+^ to LMCT during the sensitization process and low intensity emission of the lanthanide ion bands^39,40^.

Charge transfer states can sensitize lanthanide luminescence, especially in the case of trivalent lanthanide ions that can easily be reduced to the divalent state (redox-sensitive lanthanide ions), and are most often observed in Eu^3+^. In such cases, the light can be absorbed by an intense LMCT state from which the excitation energy can be transferred to the 4f-levels of the lanthanide ion. However, if the LMCT state energy is low, it will partially or totally quench the luminescence of the complex. This has been shown experimentally, where in many Eu^3+^ complexes, LMCT states constitute an important channel for depopulation of the lanthanide excited states, leading to inefficient means of sensitizing the metal center^41–45^.

The quantum yield of the 430 nm band was determined to be 9%, which is low, and points towards poor absorbed energy transfer from the ligand triplet state to LMCT. The lifetime of the 430 nm band was determined to be 8.2 ± 0.2 ns. UV–visible absorbance spectra of 2-methoxyterephtalic acid revealed absorption bands at 257 and 305 nm (Fig. S12), which are attributed to the ligand-centered π → π* and n → π* transitions respectively (n represents the “non-bonding” orbital of the carboxyl and methoxy group O atom). For EuBDC-OMe, these π → π* and n → π* transition bands were blue shifted to 254 and 297 nm, respectively (Fig. S12), likely due to carboxyl group coordination with Eu^3+^, as well as a new, broad absorbance band at 330–430 nm assigned to LMCT^17,46^. As a result of poor energy transfer between the ligand’s triplet state and excited lanthanide state, the overall intensity of fluorescence and quantum yield was low, which may decrease the sensing range and thus limit potential sensor applications, compared to MOFs with better realized antenna effected and high intensity lanthanide emission.

### pH sensing

**Figure 3:**
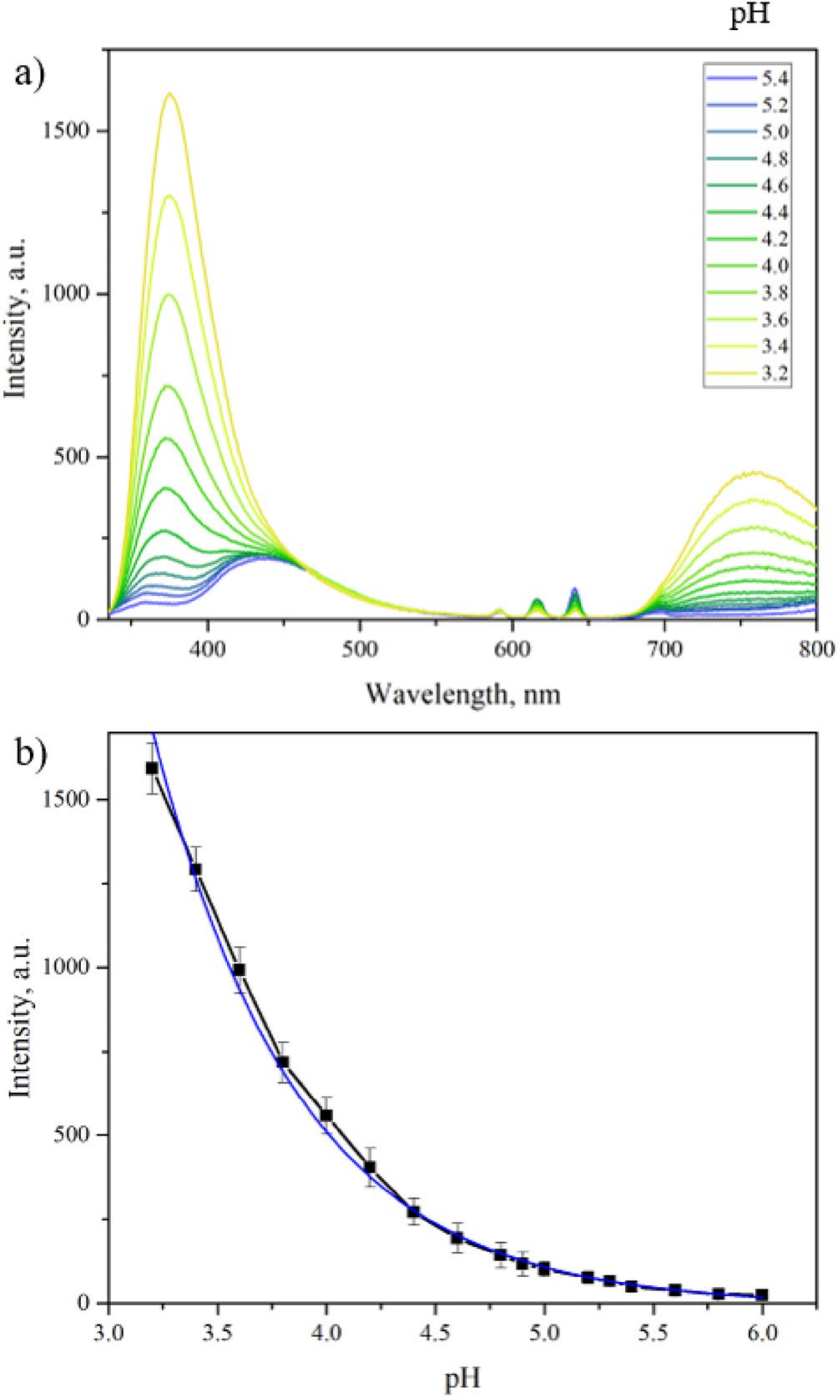
Fluorescence intensity changes (λ_exc_ = 320 nm) of EuBDC-OMe MOF particle suspensions in water at acidic pH: (**a**) fluorescence emission spectra changes with decrease in pH; (**b**) peak 375 nm intensity change corresponding to decrease in pH (MOF suspension in water pH = 5.4). Error bars represent ± standard deviation (n = 3).

Xu et al*.* reported that the ratio of the MOF’s emission bands at 375 and 430 nm could be utilized for selective ratiometric sensing of Fe^3+^ ion^23^. In our study, adding incremental amounts of FeCl_3_ to the EuBDC-OMe suspension in water resulted in an increase in the intensity of the emission band at 375 nm and a decrease in the intensity of the emission band 430 nm, which is consistent with the original report. However, our sensing experiments revealed yet another crucial observation not reported in the earlier work; the band at 375 nm increases due to changes in the pH, irrespective of the Fe^3+^ ion concentration (Fig. 3).

EuBDC-OMe suspensions prepared in varying pH solutions (< 5.4) resulted in significant changes in the 375 nm band of EuBDC-OMe (Fig. 3). This band shows an exponential increase in intensity as the pH of the solution decreases in the range between pH 6 and 3.2. Quantitatively, this relationship can be expressed using an exponential equation: $\mathrm{I}=301972{\mathrm{e}}^{-1.598\mathrm{pH}}$ (R^2^ = 0.995). Indeed, when Fe^3+^ ions were incrementally added to an already acidified suspension (pH 4.00 ± 0.02), the intensity increase of the 375 nm band was no longer observed (Fig. S13).

**Figure 4:**
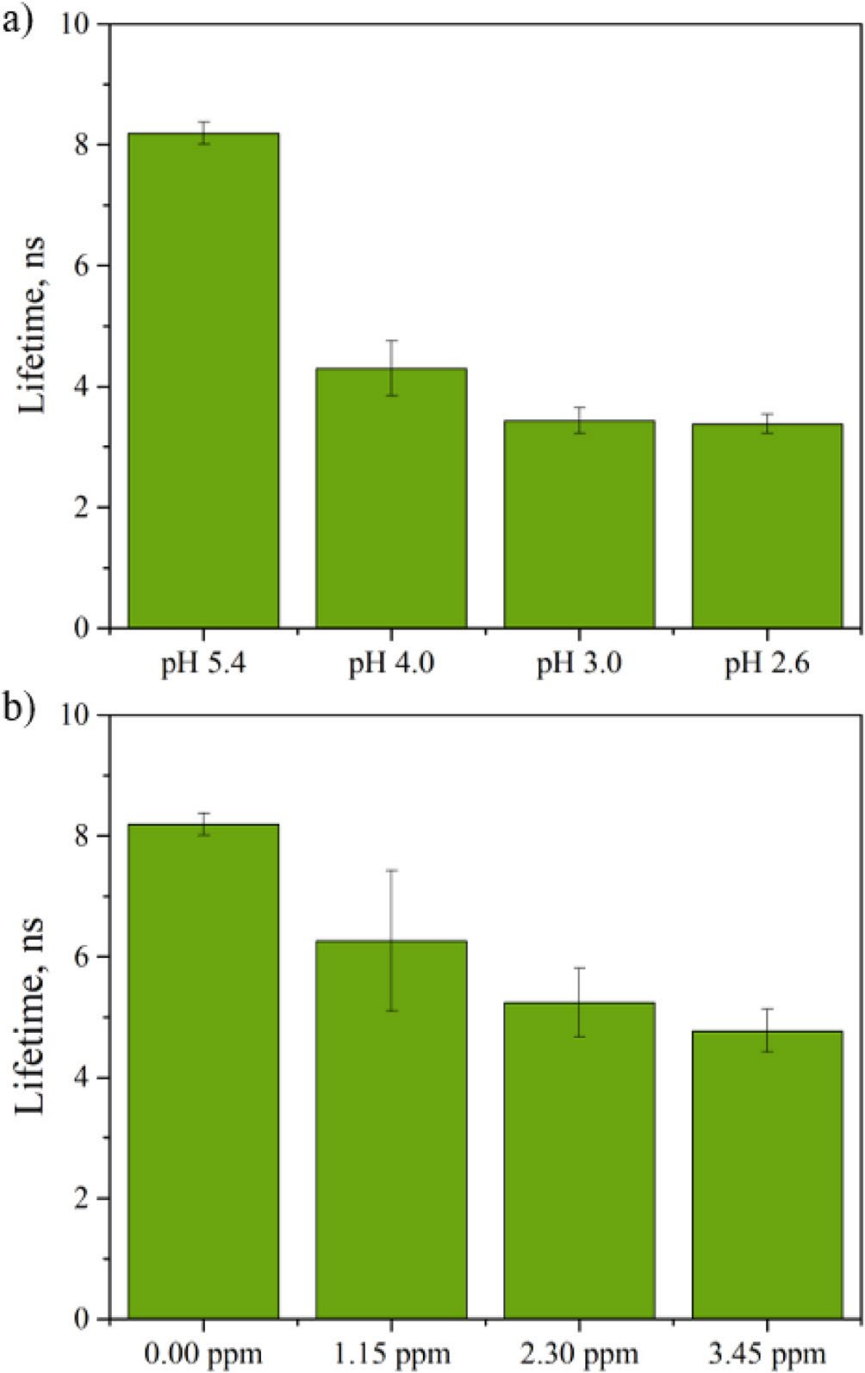
(**a**) Fluorescence band 430 nm average lifetime values at pH 2.6, 3.0, 4.0 and 5.4; (**b**) Fluorescence band 430 nm average lifetime values change with increase in Fe^3+^ ion concentration (0–3.5 ppm). Error bars represent ± standard deviation (n = 5).

Ligand protonation at the exterior of the MOF particles and changes in energy transfer between the ligand and Eu^3+^ can explain the pH sensing mechanism in EuBDC-OMe. Some lanthanide based MOFs have shown a turn-on response to a decrease in pH based on non-coordinated carboxyl groups of ligands being protonated at low pH, while deprotonating when pH is high^40,47,48^. Due to the deprotonation of the carboxylic acid functionality at higher pH, the electron density in the aromatic ring of the ligand increases, which causes an increase in the triplet excited state energy from protonated to deprotonated ligand. Changes in the triplet excited state energy, can decrease the efficiency of the antenna effect, and result in energy back transfer from a lanthanide resonance level to a ligand triplet state, as well as weakening of ligand—lanthanide ion bonding^45,49,50^. As a result, a decrease in fluorescence intensity of the lanthanide ion characteristic bands (Fig. S14) and lifetime of the ligand to metal charge transfer band (430 nm) (Fig. 4) is observed.

For EuBDC-OMe, it is expected that ultrasound treatment of the bulk crystals exposes non-coordinating carboxyl groups at the surface of the resulting nanoparticles, where the bonds between BDC-OMe and Eu^3+^ are broken^40^. The clear decrease in the lanthanide band fluorescence intensity as the pH decreases, indicates energy back transfer—repopulation of the 2-methoxyterphtalic acid triplet state from Eu^3+^ excited states. Furthermore, this process explains the change in the emission spectra; bands 375 nm and LMCT merging, and the exponential increase of the ligand band 375 nm intensity show not only increase in the total energy within the MOF, but also energy transfer from LMCT and Eu^3+^ to the ligand. This is further confirmed by the decrease in fluorescence lifetime of the band 430 nm (Fig. 4, Fig. S15c), from 8.2 ± 0.2 ns at pH 5.4 (EuBDC-OMe suspension in water) to 3.4 ± 0.2 ns at pH 3.0 (HCl). The energy back transfer hypothesis is also supported by UV–Vis absorbance spectra of EuBDC-OMe in an acidic environment (Fig. S16b).

Therefore, two competing processes contribute to fluorescence changes observed during Fe^3+^ ion sensing. Adding Fe^3+^ salts to the EuBDC-OMe suspension lowers the pH of the system, which alters the intensity of the 375 nm band due to the turn-on response to that pH decrease (Fig. S17), which can (and has been) erroneously attributed to a response of the MOF to the increased Fe^3+^ ion concentration. The 375 nm band turn-on response to a decrease in pH makes this band unsuitable for use in a ratiometric sensing paradigm. Instead, the sensing of Fe^3+^ ions by EuBDC-OMe can only be accomplished through the 430 nm band, which points towards EuBDC-OMe behaving as a turn-off sensor.

### Fe^3+^ ion sensing

**Figure 5:**
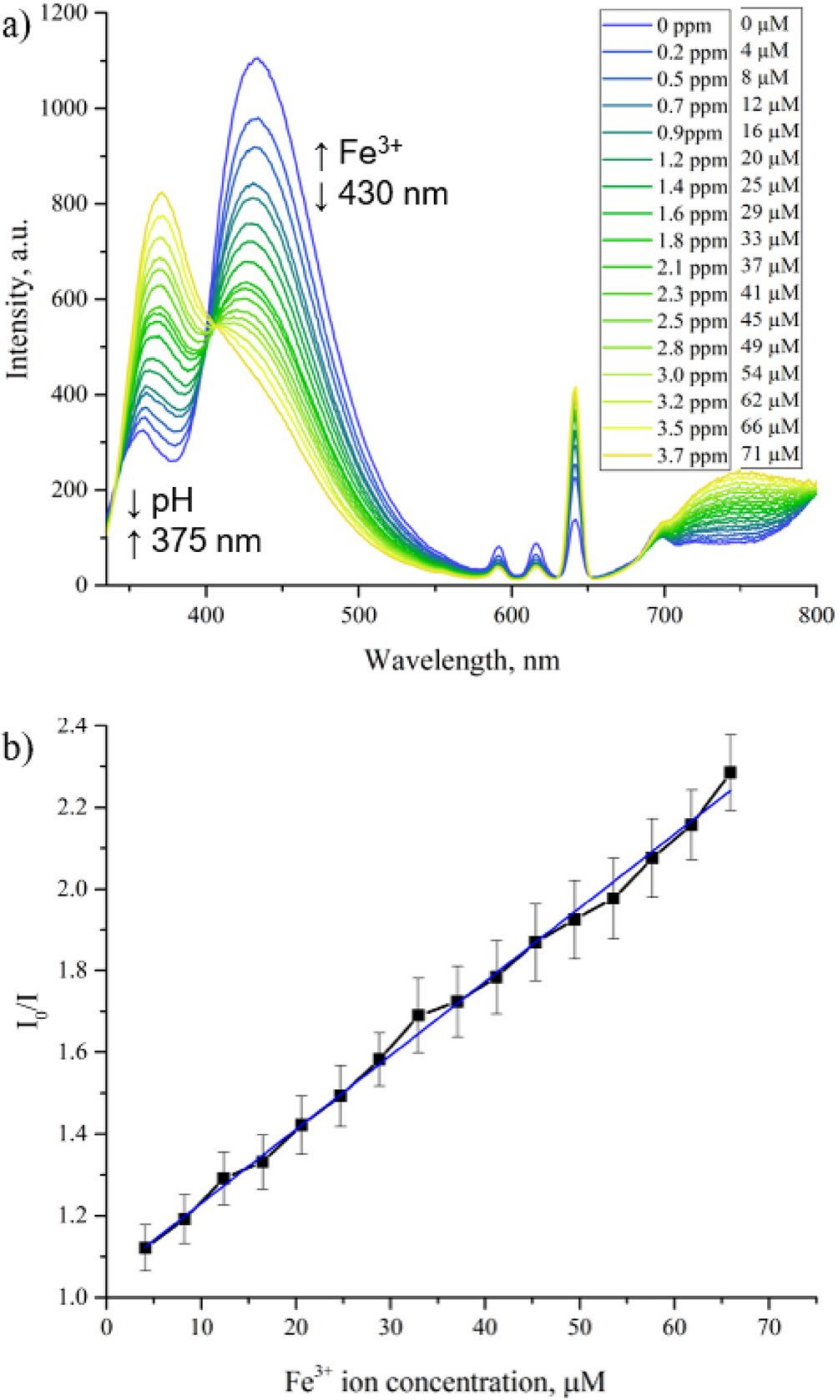
Fluorescence emission spectra (λ_exc_ = 320 nm) of EuBDC-OMe with different Fe^3+^ ion concentrations in ultrapure water: (.) concentration in ppm and molarity. Fe^3+^ causes fluorescence quenching of the band 425 nm, while band 375 nm increases as a result of pH changes; (**b**) Stern–Volmer plot of EuBDC-OMe band 430 nm quenched by Fe^3+^ ions. Error bars represent ± standard deviation (n = 5).

In the paper by Xu et al*.*^23^, the Fe^3+^ ion sensing mechanism was attributed to the electron transfer and the binding between Fe^3+^ ions and the –OH functional groups of the MOF. This conclusion was made based on fluorescence lifetime measurements and analysis of XPS oxygen 1s spectra of the MOF when exposed to Fe^3+^ ions. However, given the incorrect assignment of the MOF structure, this mechanism requires revision. In the revised structure, the methoxy functional groups (arising from 2-methoxyterepthalate) significantly diminish the available pore volume, making diffusion and binding of Fe^3+^ ions within the MOF unlikely. Therefore, we hypothesize that the sensing mechanism of the MOF relies on surface interactions between Fe^3+^ ions and the exterior the MOF crystals. As discussed above, only the 430 nm band, assigned to LMCT, responds to the Fe^3+^ ion concentration increase by decreasing in intensity, making EuBDC-OMe a ferric ion turn-off sensor (Fig. 5a). Therefore, we performed a fluorescence titration with Fe^3+^ ions and calculated the quenching coefficient K_D_ with the Stern–Volmer equation I_0_/I = 1 + K_D_[Q], where I_0_ and I are the luminescent intensity at 430 nm in the absence and presence of Fe^3+^ ions, K_D_ is the dynamic quenching constant, [Q] is the quencher (Fe^3+^) concentration (Fig. 5b)^51^. The quenching constant calculated for Fe^3+^ ions is ${K}_{D}=1.8\times {10}^{4}$ M^−1^ (concentration adjusted from µM to M), which reveals a strong quenching effect on the EuBDC-OMe luminescence. The detection limit determined experimentally from the emission spectra is 0.16 ppm or 2.9 µM Fe^3+^ions, which is higher than the detection limit determined by Xu et al*.* (1.17 µM)^23^.

The sensing range of EuBDC-OMe was found to be affected by the sonication time and particle size, indicating that the sensing mechanism indeed depends on the Fe^3+^ ion-MOF interactions at the surface of the crystals. When the sonication time was increased from 60 to 150 min, the Fe^3+^ ion sensing range was expanded by 2.5 ppm, a 55% increase (Fig. S18), which is consistent with surface interactions between the ferric ions and the EuBDC-OMe MOF particles. The decrease in MOF particle size was confirmed with SEM imaging (Fig. S19). After 60 min of sonication, a mixture of micro-particles (0.5–2 µm in diameter) and nanoparticles (100–300 nm in diameter) were found in the dried suspension. Samples from 90 min onwards contained mostly nano-sized particles (< 100 nm) and their agglomerates.

In the paper by Xu et al*.*, the authors also investigated the sensing selectivity of the MOF towards several different metal ions and reported that no other cations have a significant effect on the ratio of the 375 nm and 430 nm bands. However, since Fe^3+^ ion sensing can only be performed using the 430 nm band, we re-assessed the quenching properties of the MOF towards other metal ions. As expected, the 375 nm band (which responds to pH changes) was not significantly affected by most metal ions tested (Fig. S20a).

Fe^2+^ and Cu^2+^ ions were found to affect the intensity of the 430 nm band, however the quenching effect of these cations was significantly lower than Fe^3+^ ions (Fig. S20b). For a practical Fe^3+^ ion sensor, the ability to discern between Fe^2+^ and Fe^3+^ ions is particularly important^2^. The emission spectra of EuBDC-OMe with varying amounts of Fe^2+^ ions revealed that increasing amounts of Fe^2+^ ions quenched the intensity of the 430 nm and Eu^3+^ bands, albeit to a much lower degree than Fe^3+^ ions (quenching of 15% for Fe^2+^ ions versus 60% for Fe^3+^ ions at 75 µM), and the quenching plateaued at 0.55 ppm or 10 µM of Fe^2+^ ions (Fig. S21). Fe^3+^ ion sensing by EuBDC-OMe with Fe^2+^ ions present in the solution was also investigated (Fig. S22). In this experiment, the initial 430 nm band intensity was measured after addition of 1.8 ppm of Fe^2+^ ions, followed by incremental additions of Fe^3+^ ions. The linear relationship between Fe^3+^ ion concentration and the 430 nm band intensity was maintained, and therefore, with slight adjustments, EuBDC-OMe can still be used as a sensor to discriminate between Fe^3+^ ions and Fe^2+^ ions in the solution.

### Mechanism for the detection of Fe^3+^ ions

PXRD measurements of EuBDC-OMe confirmed that fluorescence quenching in the presence of Fe^3+^ ions is not associated with changes to the MOF structure (Fig. S23). The EuBDC-OMe Stern–Volmer plot is linear (Fig. 5b), indicating a single class of fluorophores that are all equally accessible to the quencher, and points toward a dynamic quenching mechanism^51^.

However, a linear Stern–Volmer plot does not prove that dynamic quenching of fluorescence has occurred. The measurement of fluorescence lifetimes is the most definitive method to distinguish static and dynamic quenching, as the lifetime is affected by dynamic quenching only^51^. Lifetime measurements were performed using a time domain fluorescence lifetime measurement method. The measured Opolette laser pulse decay at 320 nm, as well as the measured fluorescence curves of the EuBDC-OMe fluorescence (excited at 320 nm) are shown in Fig. S15a. The Fe^3+^ ion effect on the 430 nm decay lifetime curves can be seen in Fig. S15b, while Fig. 4b shows the changes in the average lifetime values. With increasing Fe^3+^ ion concentration in the suspension, the fluorescence lifetime gradually decreases, indicating dynamic fluorescence quenching, a process that interferes with emission from the excited state of a fluorophore after the excited state has formed^51,52^.

To help further distinguish between static and dynamic quenching, examination of the absorbance spectra of the EuBDC-OMe in the presence of Fe^3+^ ions was performed. Collisional quenching only affects the excited states of the fluorophores, and thus no changes in the absorbance spectra are expected. In contrast, ground-state complex formation will frequently result in changes of the absorbance spectrum of the fluorophore^51^. In the absorbance spectra of EuBDC-OMe with incremental additions of 1 ppm Fe^3+^ ions (Fig. S16a), the LMCT band between 330 and 430 nm show a slight change in shape, while ligand-centered π → π* and n → π* transition absorbances increase slightly with rising Fe^3+^ ion concentration. These changes can be explained by the slight decrease in pH due to the acidic nature of FeCl_3_ solutions, resulting in ligand protonation and energy back transfer from Eu^3+^ and LMCT to ligand. No significant changes attributable to the presence of Fe^3+^ ions can be observed, which further supports a dynamic sensing mechanism.

Examination of the emission spectra reveals that Fe^3+^ ions not only quench the LMCT band intensity, but also the Eu^3+^ bands. Many d-block transition metals quench luminescence of Eu^3+^ since the tails of the d–d absorption bands often have a lower energy than the ^5^D_0_ excited state of Eu^3+^. However, the energy of the lanthanide d–d transitions is strongly influenced by the ligand field, and therefore, transition metal ions can quench Eu^3+^ luminescence through interactions with the ligand as well. Indeed, the transition metals Fe^3+^, Co^3+^, Ni^2+^ and Cu^2+^ are known to be efficient quenchers for Eu^3+^ luminescence^43^.

It should be noted that Xu et al*.* used XPS to infer information about the mechanism of quenching^23^. In that work the O 1s photoemission peak was recorded for the as synthesized MOF and also after exposure to a Fe^3+^ ion solution. The authors attempted to fit the peak envelope with only two components (hydroxyl oxygen and carboxyl oxygen). Upon exposure to a solution containing Fe^3+^ ions, the profile was altered, with a higher binding energy tail that resulted in a peak fit for the hydroxyl oxygen component that had shifted to higher binding energy. The authors attributed this to a direct interaction between the MOF and the metal ion, allowing them to posit that the quenching mechanism involved electron transfer, facilitated by the complexation they inferred from the peak shift.

We recorded X-ray photoelectron spectra of our EuBDC-OMe MOF, also as-synthesized and exposed to an Fe^3+^ ion solution. Our fit for the O 1s envelope includes a necessary third component due to trapped/hydration water of the MOF, which can be seen to be the source of the higher binding energy tail that was observed in the work of the previous authors. This peak component, when correctly placed in the envelope, results in the hydroxyl oxygen peak not shifting significantly (SI, Fig. S24, Table S4) upon Fe^3+^ ion exposure (and within the likely uncertainty of the peak position determination: ± 0.2 eV). Our data and analysis do not support the mechanism highlighted by the earlier work. In terms of the mechanism of quenching, the possibility of electron transfer cannot be ruled out as occurring on the surface of the MOF crystals. An energy transfer diagram demonstrating the dynamic quenching mechanism involving electron transfer from EuBDC-OMe to Fe^3+^ can be seen in SI, Fig. S25, along with photographs of the emission of a MOF suspension illuminated with 320 nm light.

## Conclusions

An Eu^3+^ based MOF was synthesized according to a published method, and found to have a different structure and properties to that previously reported. Our studies revealed that the reaction between 2-methoxyterephtalic acid and Eu(NO_3_)_3_ produces crystals of EuBDC-OMe—a MOF composed of 2-methoxyterephtalate, and not 2-hydroxyterephtalate, as originally reported by Xu et al. SCXRD and PXRD analysis revealed that the same 3-D framework was produced, albeit with a diminished accessible surface area and smaller pore apertures, due to the presence of methoxy substituents that were previously overlooked by the original authors.

The correct structure of EuBDC-OMe explains both the presence and intensity of the fluorescence bands originating from 2-methoxyterephtalate, as well as the poor sensitization of the Eu^3+^ ions. The weak band at 375 nm originates from the ligand 2-methoxyterephtalate, while the presence of the strong band at 430 nm and low intensity Eu^3+^ bands at 590–700 nm can be explained by ligand to metal charge transfer state, resulting in a weak antenna effect within the MOF structure.

Given that the sensing properties of this material were originally attributed to an impossible OH–Fe^3+^ interaction with the MOF, we also revised the Fe^3+^ sensing properties of the system. We found that EuBDC-OMe behaves as a dual pH sensor (band at 375 nm) and turn-off sensor for Fe^3+^ ions (band 430 nm), instead of a ratiometric Fe^3+^ ion sensor. The band at 375 nm showed an exponential increase in intensity with decrease in pH, while the band at 430 nm showed a linear fluorescence quenching with increasing concentration of Fe^3+^ ions. Fluorescence lifetime measurements indicated a dynamic quenching mechanism for Fe^3+^ ion sensing, where the LMCT band at 430 nm interferes with the emission of the MOF after the excited state has formed. Therefore, EuBDC-OMe is a feasible ‘turn-off’ sensor for Fe^3+^ which operates based on surface interactions. Our work also highlights the need for careful structural assignment and consideration of environmental effects (e.g., pH) in attributing the sensing properties of lanthanide MOF materials.

## Supplementary Information

Supplementary Information.

## Data Availability

The datasets used and/or analysed during the current study are available from the corresponding author on reasonable request.
