# Supplementary material for: A europium metal–organic framework for dual Fe3+ ion and pH sensing
Source: Sci Rep. 2022 Jul 14;12:11982. doi: 10.1038/s41598-022-15663-z (PMC9283444; doi:10.1038/s41598-022-15663-z)
Supplement: Supplementary file 1 — Supplementary Information. [file 41598_2022_15663_MOESM1_ESM.docx]

Supporting Information

A Europium Metal-organic Framework for Dual Fe^3+^ ion and pH Sensing.

Linda Rozenberga^1^, William Skinner^1^, David G. Lancaster^1^, Witold M. Bloch^2*^, Anton Blencowe^3^, M. Krasowska^1^, David A. Beattie^1*^.

^1^ Future Industries Institute, University of South Australia, Mawson Lakes, South Australia 5095, Australia.

^2^ Department of Chemistry, University of Adelaide, Adelaide SA 5000

^3^ Applied Chemistry and Translational Biomaterials Group, UniSA Clinical and Health Science, University of South Australia, Adelaide, South Australia 5000, Australia.

* Corresponding Authors (Email: [David.Beattie@unisa.edu.au](mailto:David.Beattie@unisa.edu.au) and Witold.Bloch@adelaide.edu.au)

Table S1. Summary of luminescent ratiometric MOF based sensors for Fe^3+^ ions.

| MOF | Type | Year | Peak ratio | Sensing range | Selectivity | pH stability | pH sensing |
| --- | --- | --- | --- | --- | --- | --- | --- |
| EuOHBDC [1] | Lanthanide MOF | 2018 | I_375_/I_427_ | 10-50 µM | Fe^3+^ | N/A | N/A |
| Eu3+@CAU-11 [2] | Eu^3+^ post-synthesis functionalized MOF | 2019 | I_389_/I_615_ | 0.05-10 mM | Fe^3+^, Cu^2+^ |  | N/A |
| SRB@UiO-66 [3] | Fluorescent dye post-synthesis functionalized MOF | 2020 | I_480_/I_590_ | 0.1-1 mM | Fe^3+^ | 2-12 | No |
| Eu_0.07_Gd_0.03_-MOF [4] | Eu/Gd + mixed ligand MOF | 2021 | I_614_/I_415_ | 5-60 µM | Fe^3+^, ascorbic acid | 3–11 | N/A |
| [Zn_2_(OH)(1,4-ndc)_1.5_(Cz- 3,6-bpy)]·2H_2_O [5] | Mixed ligand MOF | 2021 | I_426 and_ I_482_ | 1-1 mM | Fe^3+^, Al^3+^, Cr^3+^, pH, CrO_4_^2−^ , Cr_2_O_7_^2−^ | 3-12 | Yes |
| Eu^3+^: CDs@ZIF-8 [6] | Eu^3+^ and CD post-synthesis functionalized MOF | 2021 | I_458/_ I_612_ | 1-10 µM | Fe^3+^ | N/A | N/A |


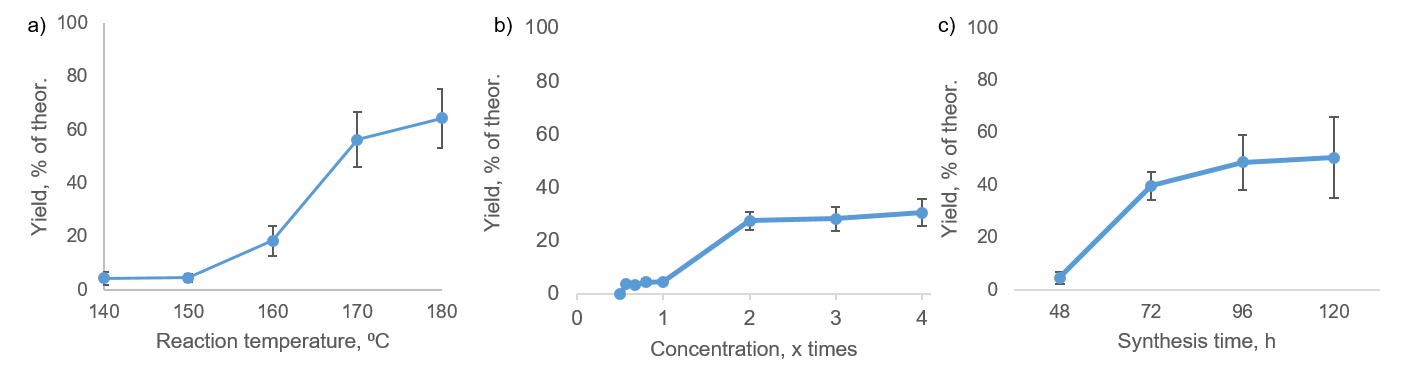


**Figure S1.** Synthesis optimization - reaction condition effect on EuBDC-OMe yield: a) reagent concentration; b) reaction time; c) reaction temperature. The original concentration, time and temperature used by Xu et al. were 1, 48 h and 140 °C, respectively.[7]

We observed that increase in synthesis temperature by 20 °C resulted in higher synthesis yield (from 4 % to 20 %), with negligible changes in PXRD pattern (**Figure S1a and S5a**). While temperature above 160 °C resulted in even higher yield, it had undesirable effect on MOF structure and PXRD pattern. Reagent concentration and reaction time effect on synthesis outcome was also investigated (**Figure S1b and S1c**). The yield was considerably increased when at least two times higher reagent concentration in water was used (preferably 3 times higher in 200 ml volume reactor), and when reaction time was extended by additional 24 h. With the three optimized parameters combined, 160 °C reaction temperature, three times higher reagent concentration in water and 72 h long synthesis, the yield was increased from 4% to 38.5%.

# X-ray crystallography

## General methods

Single crystals were mounted in paratone-N oil on a plastic loop. X-ray diffraction data for EuBDC-OMe were collected at 100(2) K on the MX-2 beamline of the Australian Synchrotron.[8] The structure was solved by direct methods using SHELXT[9] and refined with SHELXL[10] and ShelXle[11] as a graphical user interface. All non-hydrogen atoms were refined anisotropically and hydrogen atoms were included as invariants at geometrically estimated positions. X-ray experimental data is given in Table S2.

Stereochemical restraints for BDC-OMe was generated by the GRADE program using the GRADE Web Server (http://grade.globalphasing.org) and applied in the refinement. A GRADE dictionary for SHELXL contains target values and standard deviations for 1,2-distances (DFIX) and 1,3-distances (DANG), as well as restraints for planar groups (FLAT). This helped to produce a stable model of the disordered ligand. All displacements for non-hydrogen atoms were refined anisotropically. The refinement of ADP's for carbon, nitrogen and oxygen atoms was aided by similarity restraints (SIMU).[12] The contribution of the electron density from disordered, pore-bound solvent molecules, which could not be modelled with discrete atomic positions were handled using the SQUEEZE[13] routine in PLATON,[14] which strongly improved all figures of merit (FOM).

**Time domain fluorescence lifetime measurement method**

The lifetime measurements of the EuBDC-OMe were performed with and without Fe^3+^ ions present, and with various pH levels (decreased by addition of HCl). Fluorescence lifetimes were measured at 430nm as shown in Figure 1 using a monochromator, amplified silicon detector, and oscilloscope[15]. An Opotek opolette HE355 LD laser (a) was configured to produce a 320 nm emission which was filtered through a Jarrell-Ash 82-410 monochromator (b) to eliminate residual laser energy at longer wavelengths from the frequency conversion processes. The excitation beam was directed to the sample via two Thorlabs EO1 Fused Silica Broadband dielectric mirrors (C_1_ and C_2_). A Quartz cuvette (d) held 3ml of EuBDC-OMe suspension in water (0.1mg/ml). Fluorescence was collected at 90° relative to excitation and imaged into a second monochromator (g) with AR coated f = 50 mm (e) and f = 30 mm (f) lenses (Thorlabs AC254-050-A-ML). The second monochromator (Thermo Oriel 77250B Series) (g) used a 77298 Grating Assembly, had input and output slit widths of 7.35mm and 6.21mm, and was set to 430 nm (Δλ~ 6 nm). The output narrow-band fluorescence was imaged by a f = 35 mm lens (Thorlabs AC254-035-A-ML) (h) onto the Thorlabs PDA10A2 fixed gain Si amplified detector (i), (150 MHz bandwidth and 2.3 ns rise time). Detector voltage as a function of time was collected using a 1 GHz bandwidth RIGOL DS6104 digital oscilloscope (j).

Voltage as a function of time showing the exponential decay of EuBDC-OMe fluorescence at the 430nm wavelength was collected from the oscilloscope and was analysed according to a previously published method[15–18] . In short, the decay curves were normalised and plotted on a logarithmic scale. The data was then fitted to an asymptotic exponential decay function using OriginPRo2016. The lifetime value was determined as the time for the intensity to drop by 1/e or to 36.79%. Each lifetime measurement was repeated 5 times, and the final value reported as the average. The excitation pulse is less than the fluorescence lifetimes with a measured full-width at half-maximum of ~3.6 ns. The combination of a ~3.6 ns excitation pulse width, and detector bandwidth of 150 MHz (6.7 ns response time) indicates that the measured fluorescence lifetimes will be a convolution of pulse width, detector response, and fluorescence lifetime. Whilst our measured lifetimes are overestimated, their relative lifetimes are reasonably precise.

**
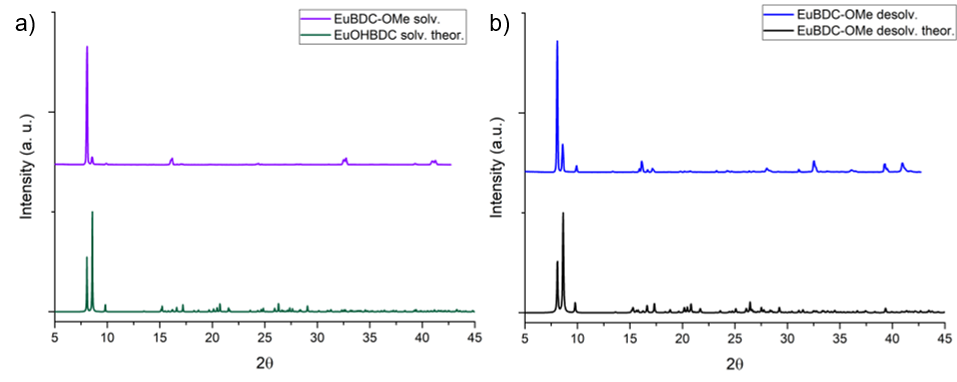
**

**Figure S2**. a) The theoretical PXRD pattern of the EuOHBDC MOF from the orginal study, in solvated (water) state versus our synthesized EuBDC-OMe in solvated state (synthesized at 140 °C); b) The theoretical PXRD pattern of the EuBDC-OMe MOF obtained in our study, in solvated (water) state versus our synthesized EuBDC-OMe in solvated state (synthesized at 140 °C)

**Table S2:** X-ray experimental data for EuBDC-OMe

| Compound | **EuBDC-OMe** |
| --- | --- |
| CCDC number | 2141230 |
| Empirical formula | C_27_H_19_Eu_2_O_17_ |
| Formula weight | 919.34 |
| Crystal system | Monoclinic |
| Space group | P2**_1_**/n |
| *a* (Å) | 11.513(2) |
| *b* (Å) | 6.8290(14) |
| *c* (Å) | 20.976(4) |
| α (º) | 90° |
| β (º) | 102.84(3) |
| γ (º) | 90° |
| Volume (Å^3^) | 1607.9(6) |
| *Z* | 2 |
| Density (calc.) (Mg/m^3^) | 1.899 |
| Absorption coefficient (mm^-1^) | 3.940 |
| F(000) | 886 |
| Crystal size (mm^3^) | 0.09 × 0.07 × 0.06 |
| θ range for data collection (º) | 1.865 to 29.023 |
| Reflections collected | 10171 |
| Observed reflections [R(int)] | 3439 [0.0359] |
| Goodness-of-fit on F^2^ | 1.059 |
| R_1_ [I>2σ(Ι)] | 0.0500 |
| wR_2_ (all data) | 0.1442 |
| Largest diff. peak and hole (e.Å-3) | 1.309 and -3.301 |
| Data / restraints / parameters | 3439 / 577 / 277 |

**
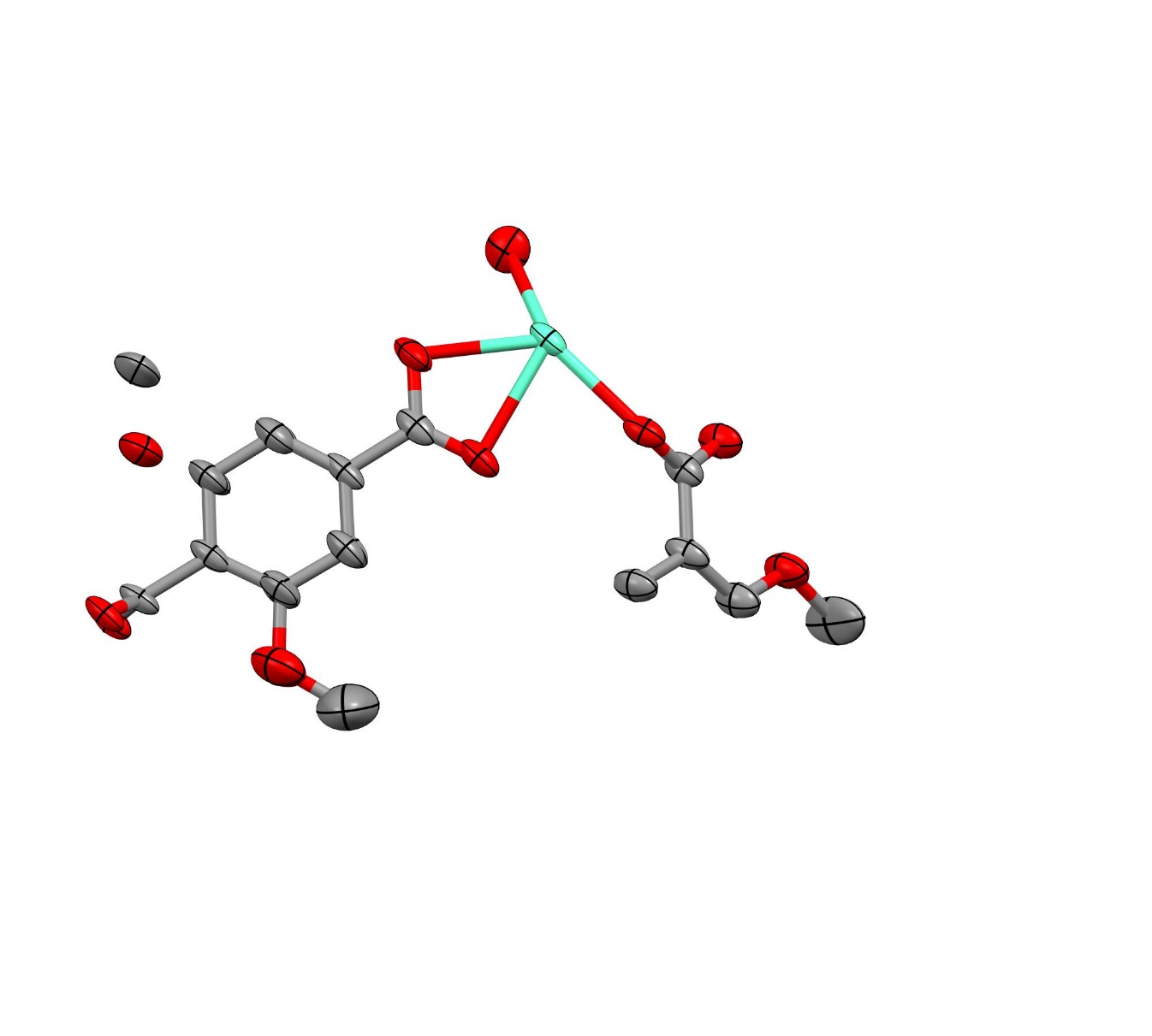
**

**Figure S3.** The asymmetric unit of the X-ray structures of EuBDC-OMe with all non-hydrogen atoms shown as ellipsoids at the 50% probability level.


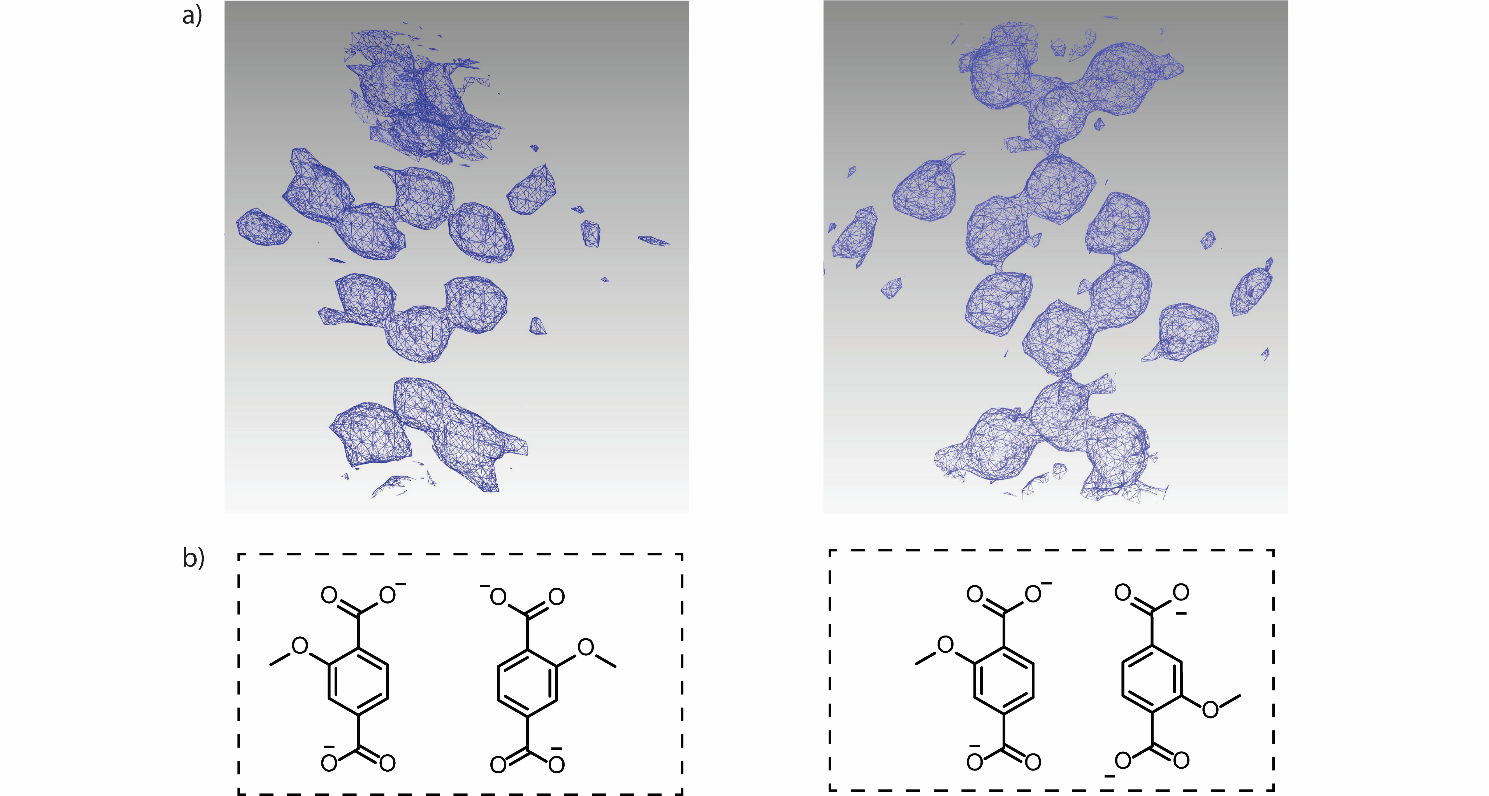


**Figure S4.** a) The F_obs_ maps of the two crystallographically unique BDC-OMe ligands clearly shows electron density corresponding to the methoxy groups; b) the relative positional and rational disorder of the ligand.


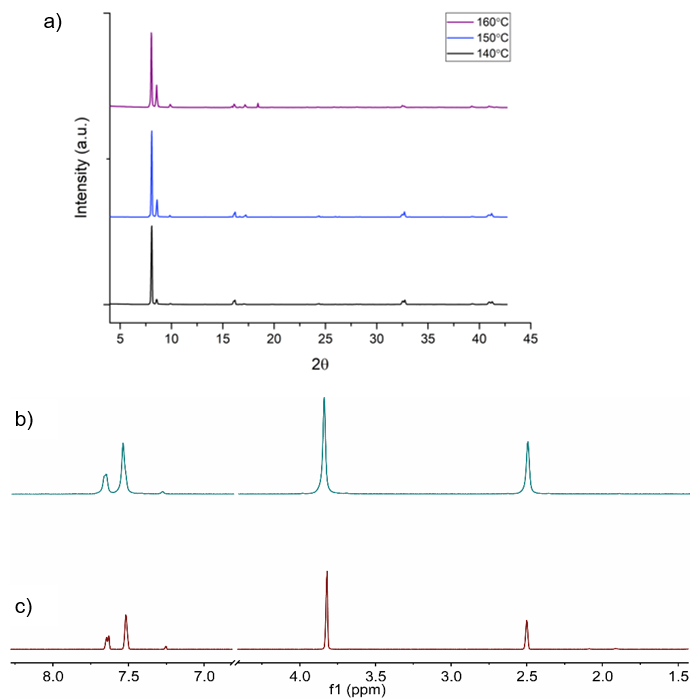


**Figure S5.** a) PXRD patterns of the EuBDC-OMe MOFs synthesised at 140, 150 and 160 °C; ^1^H NMR spectra (500 MHz, DMSO-d_6_) showing the digested EuBDC-OMe synthesised at b) 140 and c) 160 °C.

**Figure S6.** Nitrogen adsorption isotherm of EuBDC-OMe, measured at 77 K. Activation conditions: high vacuum, 90 °C for 3 h, then 65 °C overnight. BET surface = 132.9 ± 2.2 m²/g.

**Figure S7.** Derivation of the BET surface area from the 77 K nitrogen adsorption isotherms for EuBDC-OMe.

**Figure S8**. Pore size distribution, as derived from the 77 K N_2_ isotherm of EuBDC-OMe.


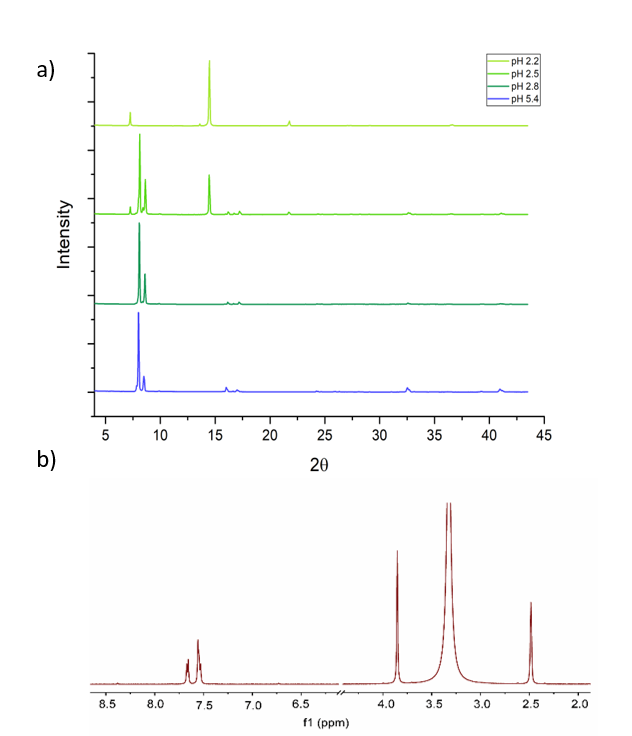


**Figure S9.** a) PXRD patterns of EuBDC-OMe in dilute HCl solutions at pH 2.2, 2.5, 2.8 and 5.4; c) ^1^H NMR spectrum (**) of EuBDC-OMe soaked in HCl at pH 2 for 24 h, then washed with ultrapure water 3 times and dried under high vacuum. NMR (500 MHz, DMSO-d_6_) was performed to identify white powder residue: 2-methoxyterephtalic acid.


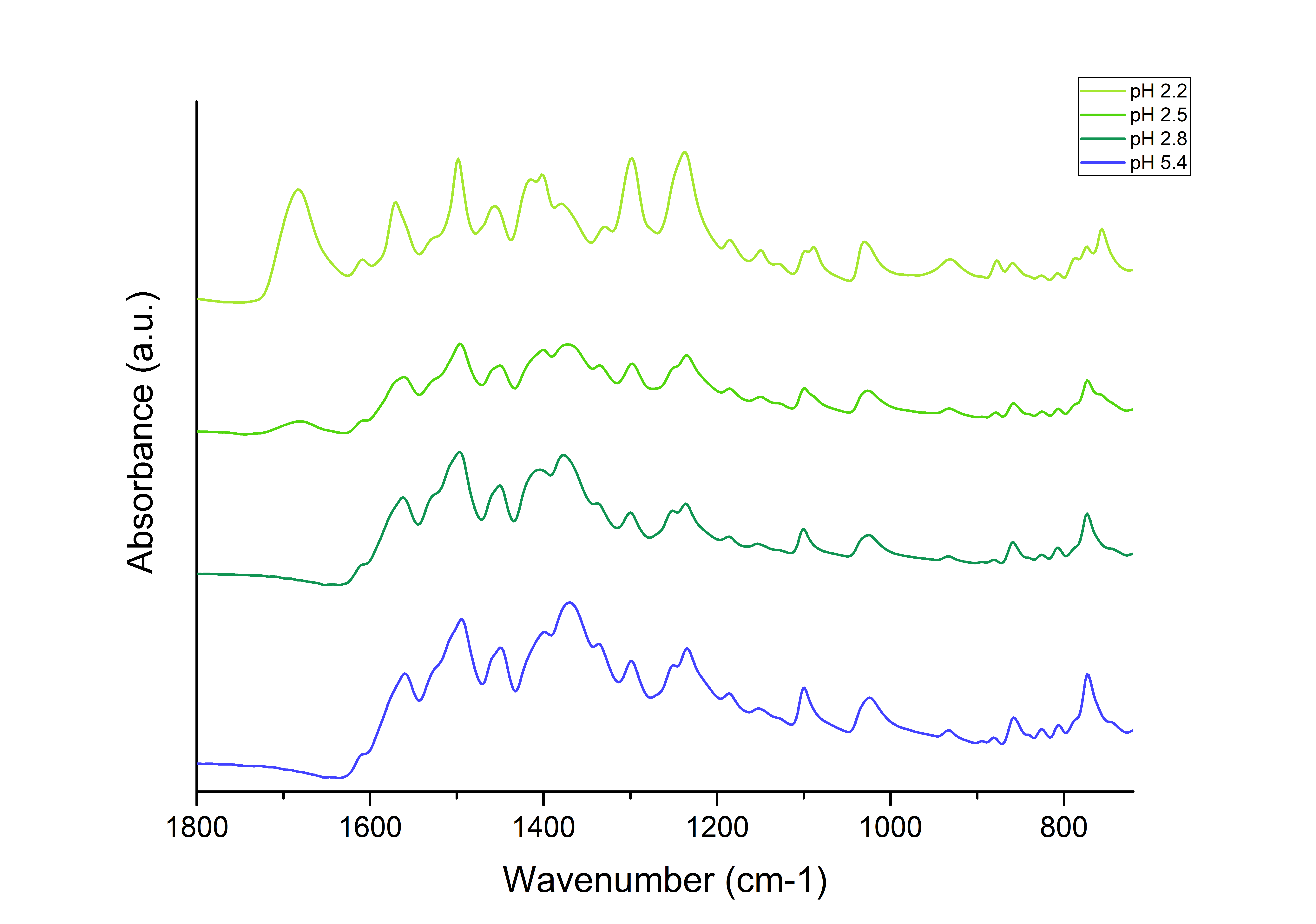


**Figure S10.** FTIR-ATR spectra showing the degradation of EuBDC-OMe below pH 2.8: emergence of the peak at 1690 cm^-1^ assigned to the ν(C=O) vibrational mode of *p*-C_6_H_4_(COOH)_2_ at pH < 2.5 indicates cleavage of bonds between Eu^3+^ and the 2-methoxyterephtalic acid ligand, and protonation of the carboxylate groups. At pH 2.2, the absorbance of the peak at 1690 cm^-1^ significantly increased, as well as the absorbance of peaks at 1300 and 1234 cm^-1^ corresponding to the i-plane bending and stretching/bending vibrations of the COH, respectively, as a result of protonation of the carboxylate groups. Peak assignment is based on literature of terephtalic acid infrared spectroscopy studies.

**Table S3**. ATR FTIR spectra peak assignment of EuBDC-OMe suspension in water.

| EuBDC-OMe suspension in water peak wavenumber (cm^-1^) | 2-methoxyterephtalic acid suspension in water peak wavenumber (cm^-1^) | Assignment |
| --- | --- | --- |
|  |  |  |
|  | 1690 | ν_as_(C=O) +δ(COH)[19] |
|  | 1611 | ν_as_(C=O)^11^ |
| 1560 | 1574 | β(CCH) + ν(C C)[19–21] |
| 1495 | 1500 | β(CCH)+ ν(C=C)^11, 12^ |
| 1470 | 1465 | –OCH_3_[22] |
|  | 1417 | ν(C=C) + δ(C=C-C) ^11^ |
| 1398 | 1402 | –OCH_3_^14^ |
| 1369 |  | C–O stretches of coordinated linkers in MOFs [21,23] |
| 1336 |  |  |
| 1300 | 1304 | δ(COH)+ ν(CO)^11^ |
| 1234 | 1244 | Stretching and bending vibrations of the C–O–H in the carboxyl group^12^ |
| 1151 | 1149 | β(CCH) + δ(COH)^11^ |
| 1127 | 1128 | β(CCH) + ν(C C)^11^ |
| 1024 | 1033 | –OCH_3_^14^ |
| 931 | 931 | ρ(CH) o.o.p^11^ |
| 858 | 850 | ρ(CH)^11^ |
| 841 | 848 | γ(CCC)φ + δ(C=C=C) ^11^ |
|  | 788 | ρ(OH) + ρ(CH)^11^ |
| 772 |  | Ring-out-of-plane vibration of the aromatic ring[21] |
|  | 756 |  |
| 695 | 688 | δ(C C O)+ δ(C C O) γ(CCC)φ ^11^ |

Annotations: ν – stretching vibration, ν_as_ – asymmetric stretching vibration, ν_s_ – symmetric stretching vibration, γ – out-of-plane bending vibration, γ_r_ – out-of-plane rocking vibration, γ_w_ – out-of-plane wagging vibration, γ_t_ – out-of-plane twisting vibration, δ – in-plane bending vibration, δ_w_ – in-plane wagging vibration.


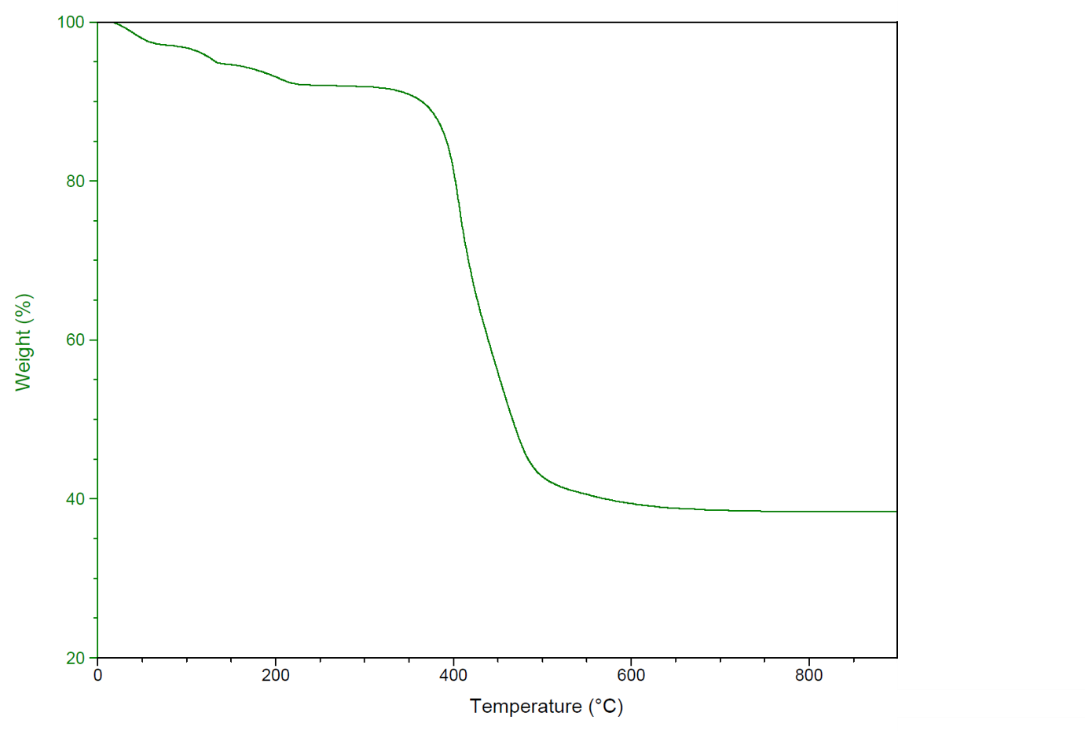


**Figure S11.** TGA results of the EuBDC-OMe MOF showed differences in the thermal stability compared to the results in the original synthesis article[7]. It was reported previously that the MOF is thermally stable up to 450 °C, however we found that EuBDC-OMe MOF has lower decomposition temperature. Synthesised and dried EuBDC-OMe shows an initial 3% weight loss 25-70 °C as a result of methanol in the MOF pores from the activation process. Following weight decrease by 4.5% at 70-220 °C corresponds to evaporation of water from the MOF pores, as well as the water bond to the crystalline structure. Lastly, the EuBDC-OMe decomposes at 330-550 °C leaving 41.2% residue, most likely Eu_2_O_3_.


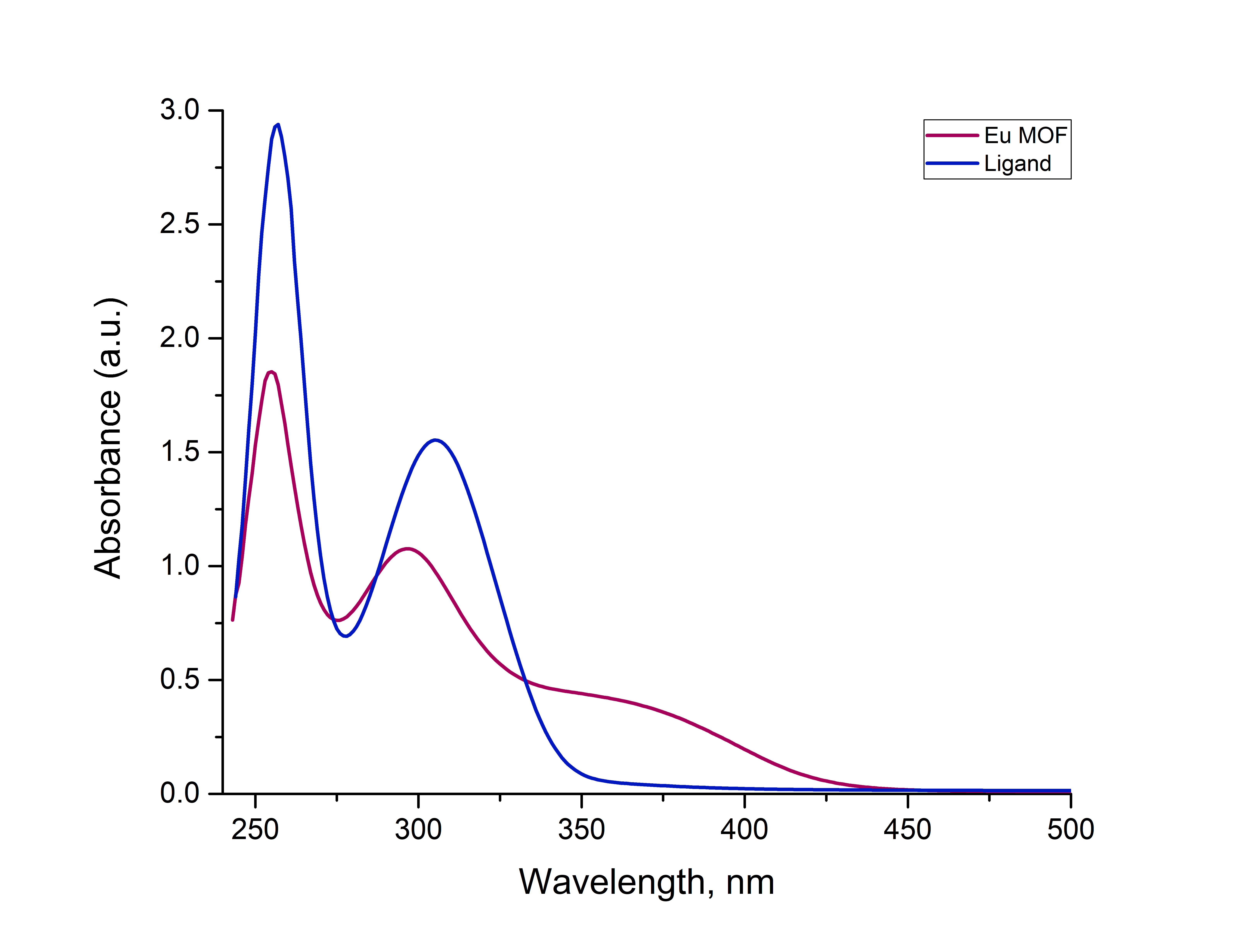


**Figure S12.** UV-visible absorbance spectra of EuBDC-OMe and 2-methoxyterephtalic acid (ligand).


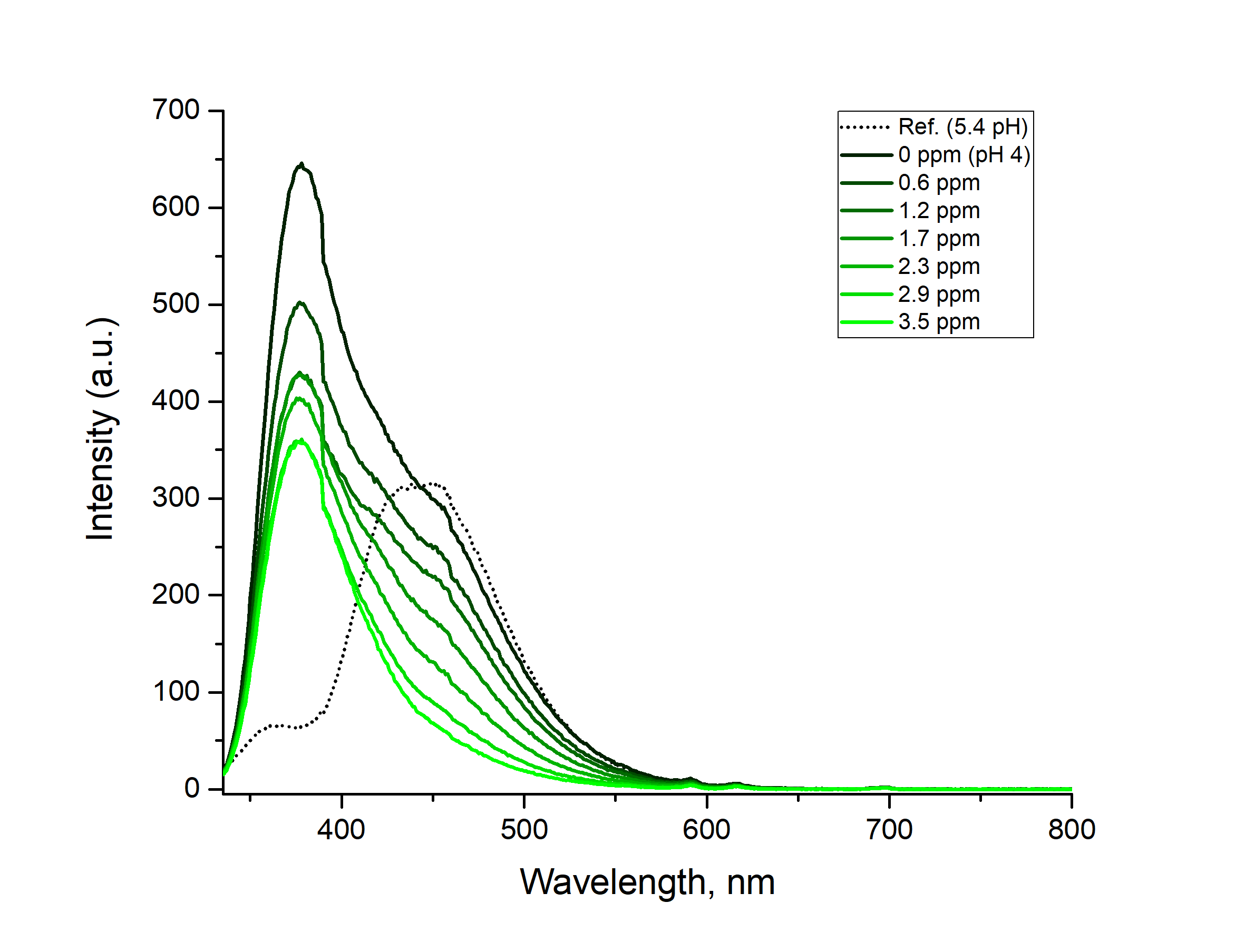


**Figure S13**. Fe^3+^ ion sensing by EuBDC-OMe suspension with pH adjusted to 4.00 ± 0.02: dotted line represents EuBDC-OMe suspension in water, solid black line - EuBDC-OMe suspension at pH 4.0 with no Fe^3+^ ions added, followed by Fe^3+^ 0.6-3.5 ppm titration spectra.

**Figure S14.** Changes in Eu^3+^ band 616 nm intensity with decrease in pH. Error bars represent ± standard deviation (n = 3). Linear equation is determined across the pH range of 2.4 to 4.2 (dark blue in figure).


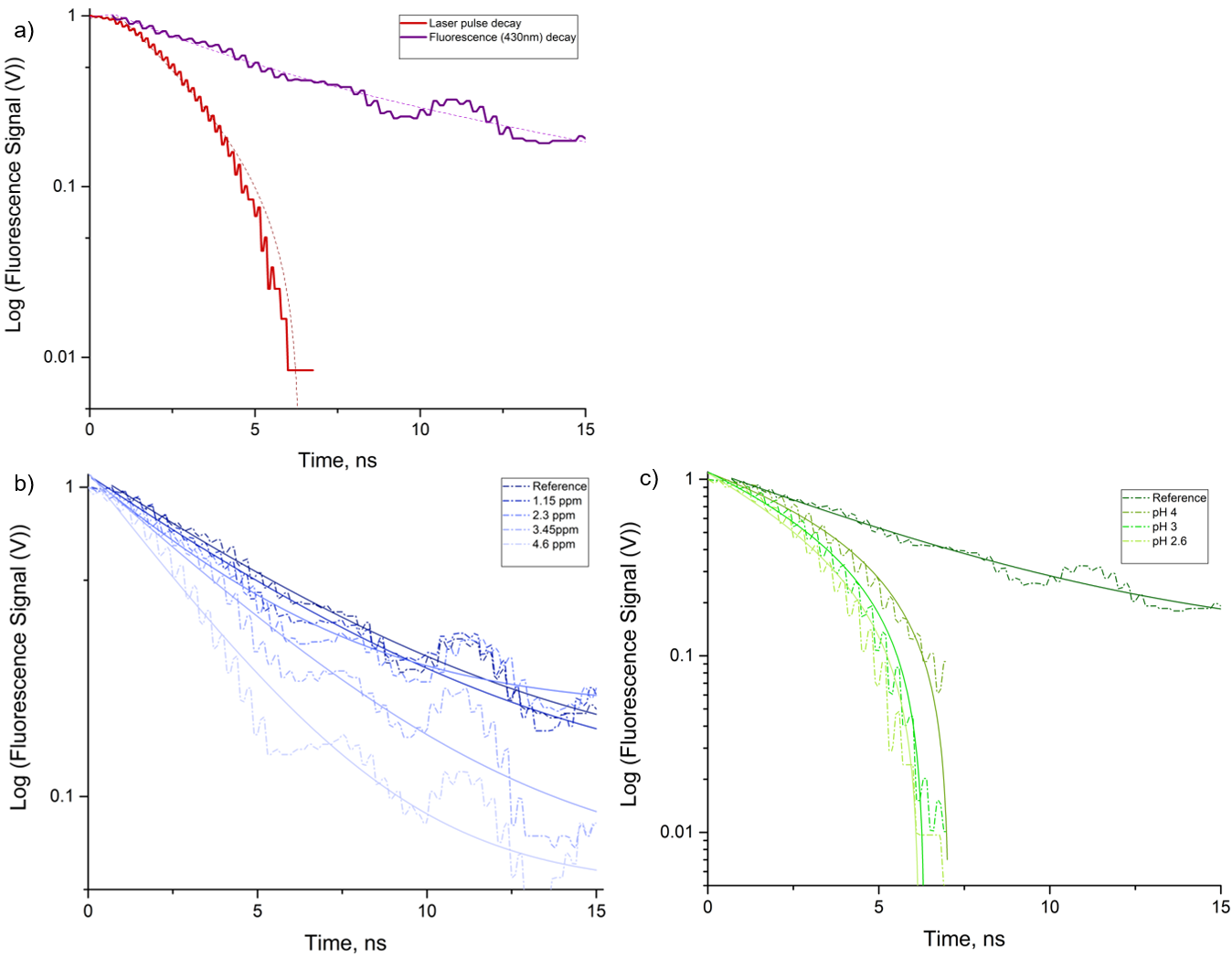


**Figure S1*5*.** a) Opolette laser pulse decay curve *vs* EuBDC-OMe fluorescence at 430 nm decay curve; b) fluorescence at 430 nm decay curve changes with increased concentration of Fe^3+^ ion; c) fluorescence at 430 nm decay curve changes with decrease in pH.


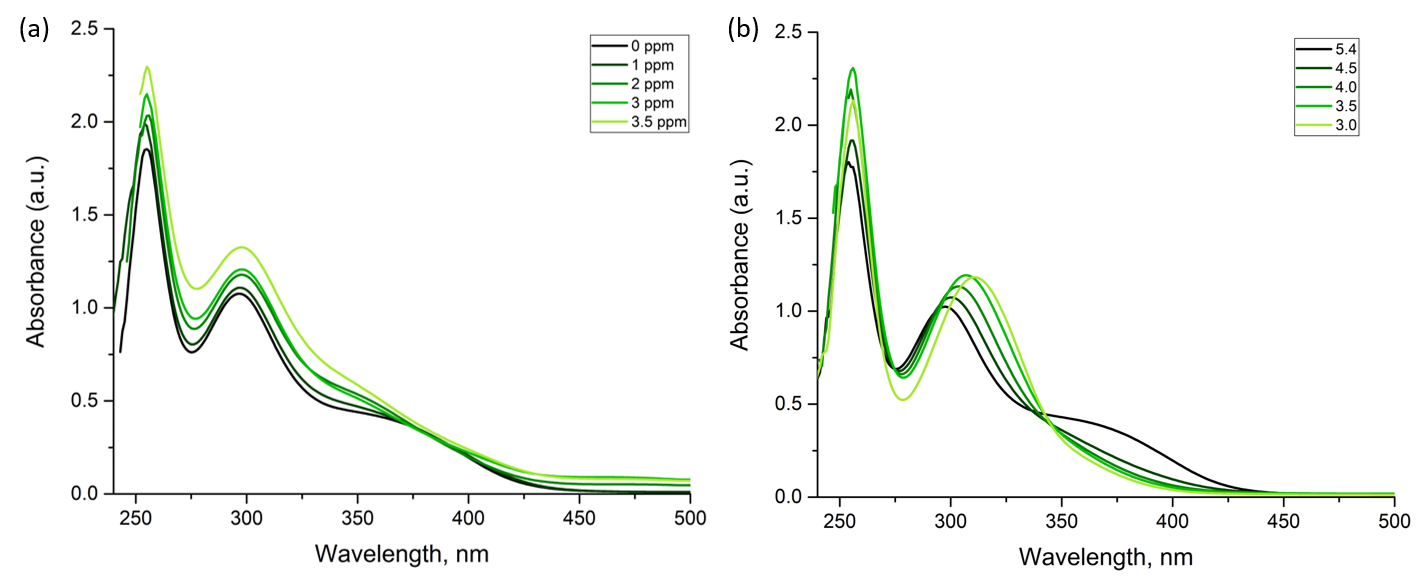
 **Figure S16**. (a) Changes in EuBDC-OMe absorbance spectra with addition of 0-3.5 ppm of Fe^3+^ ions; (b) Changes in EuBDC-OMe absorbance spectra with decrease in pH from 5.4 (Milli-Q ultrapure water) to 3 (dilute HCl).

The spectra in **Figure S17b** show the disappearance of the LMCT absorbance band between 330 and 430 nm, and an increase in the absorbance of both ligand bands at 254 and 297 nm. There is also a noticeable red shift in the n → π* absorbance band from 297 to 311 nm, beyond the free ligand n → π* absorbance band position at 305 nm, indicating changes in the “non-bonding” orbital of the carboxyl group O atom as a result of protonation. The LMCT band decrease in intensity, and increase in ligand band absorbance corresponds to energy being transferred back to ligand as a result of increasing H^+^ concentration.


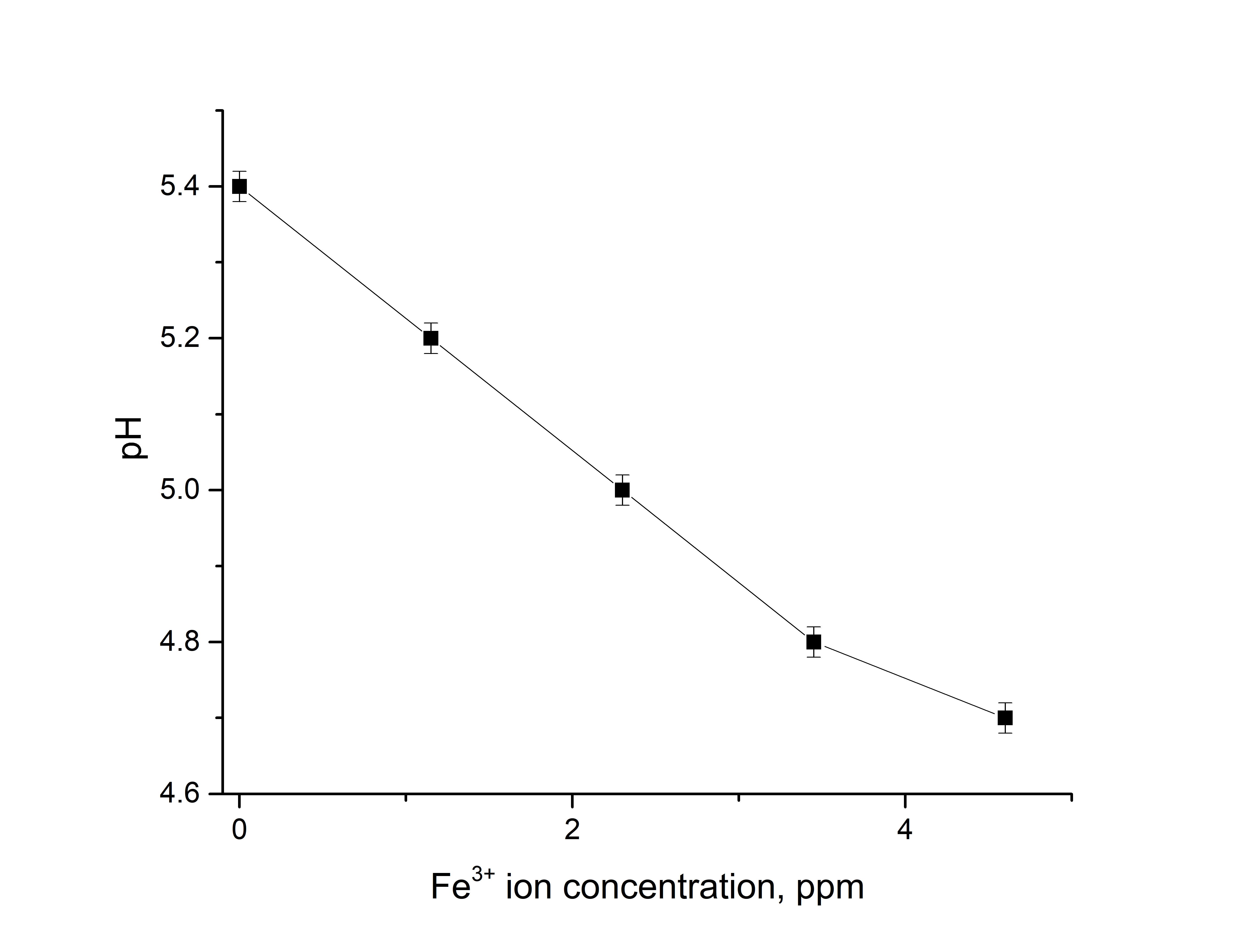


**Figure S17.** pH changes of EuBDC-OMe suspension with incremental addition of Fe^3+^ ions in the sensing range of 0-4.5 ppm (FeCl_3_ solution). Error bars represent ± standard deviation (n = 3).


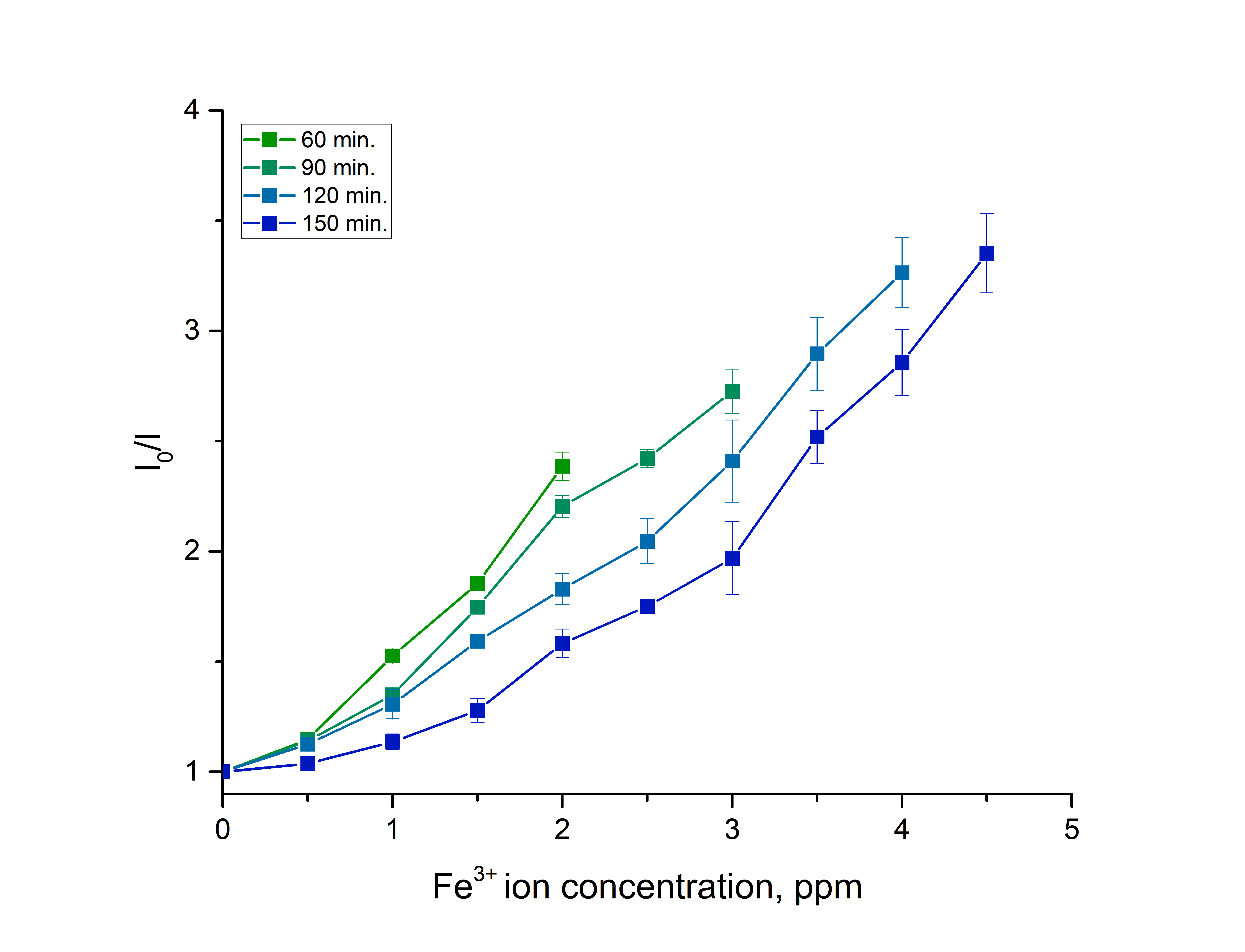


**Figure S18**. EuBDC-OMe sensing range of Fe^3+^ ions changes with increasing suspension sonication time. Error bars represent ± standard deviation (n = 3).


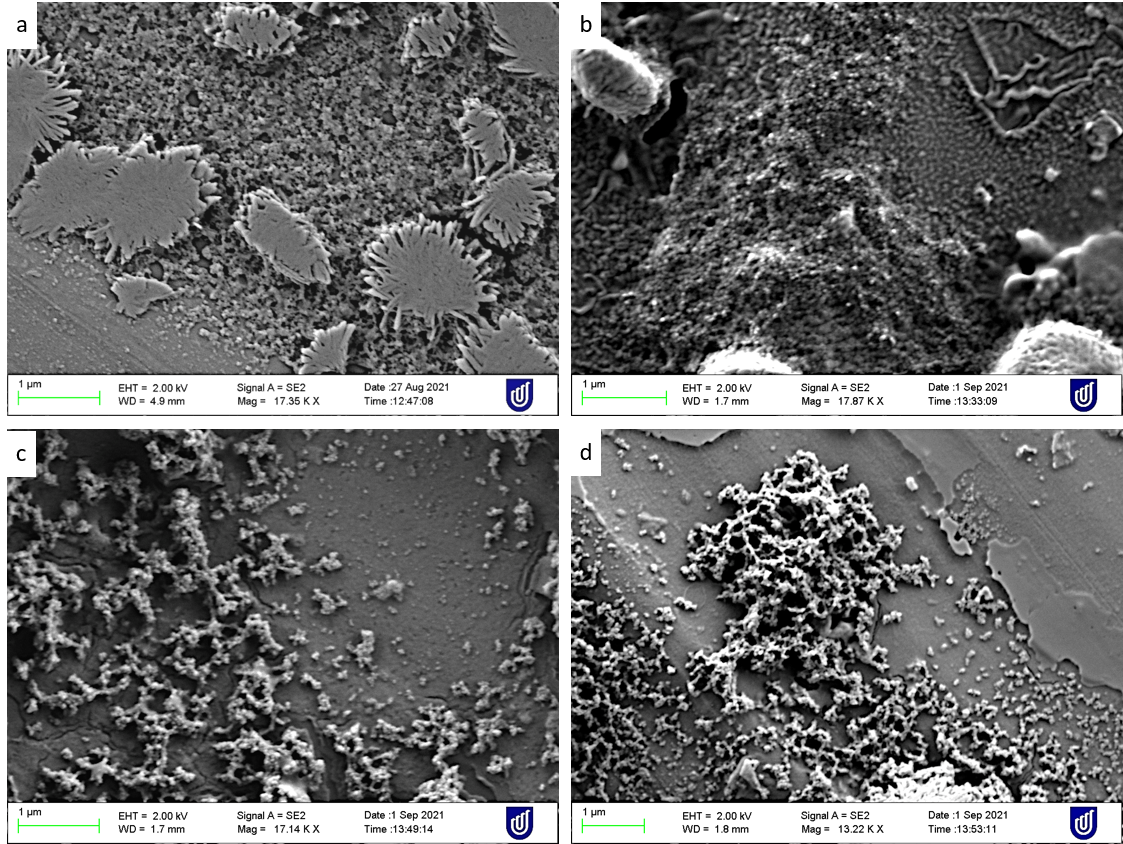


**Figure S19.** SEM images of EuBDC-OMe after different sonication times: a) 60 min; b) 90 min; c) 120 min; d) 150 min. More accurate measurements of the particle sizes and their fraction % estimation could not be performed with microscopy due to their small size, or with particle sizing techniques like Zeta-Sizer, due to the EuBDC-OMe fluorescence.


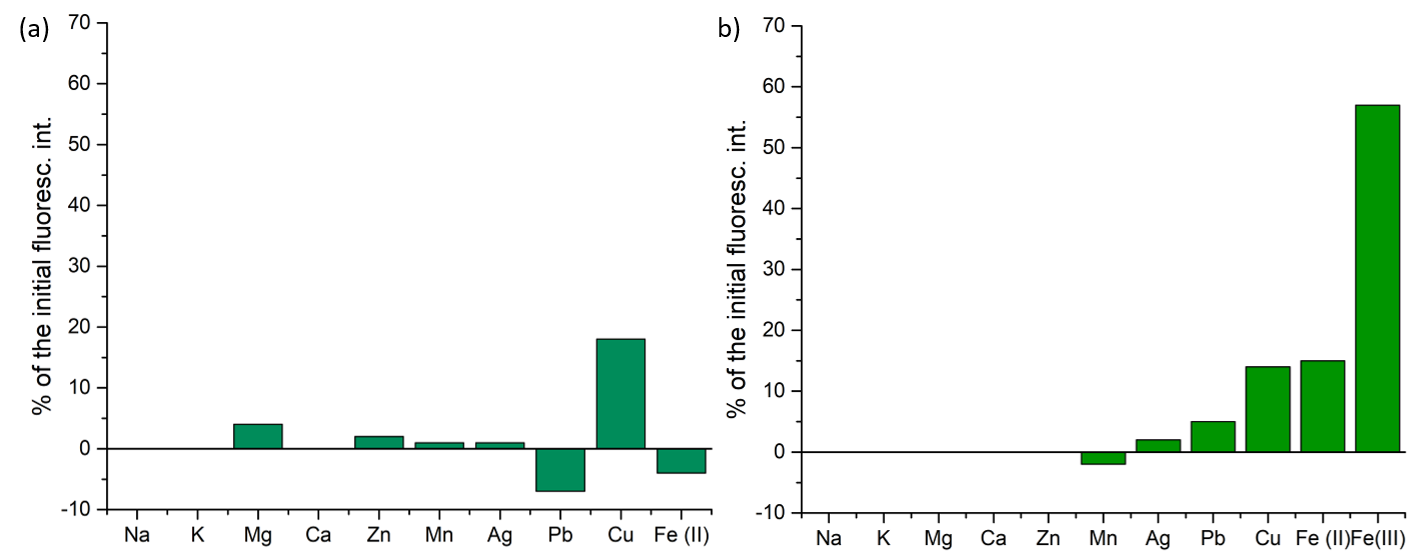


**Figure S20.** Cation selectivity of EuBDC-OMe: band a) 375 and b) 430 nm sensitivity towards different metal cations (75 µM).


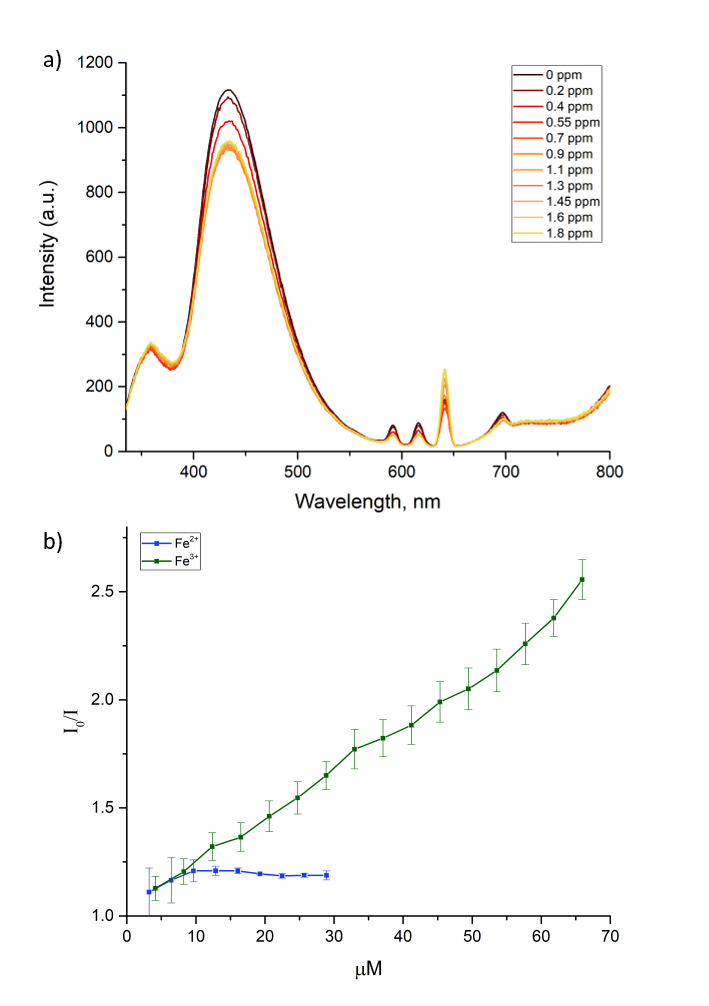


**Figure S21.** a) Fluorescence emission spectra of EuBDC-OMe with different Fe^2+^ concentrations in water (λ_ex_ = 320 nm); b) a relationship curve of the luminescence intensity versus Fe^2+^ / Fe^3+^ concentration. Error bars represent ± standard deviation (n = 3).


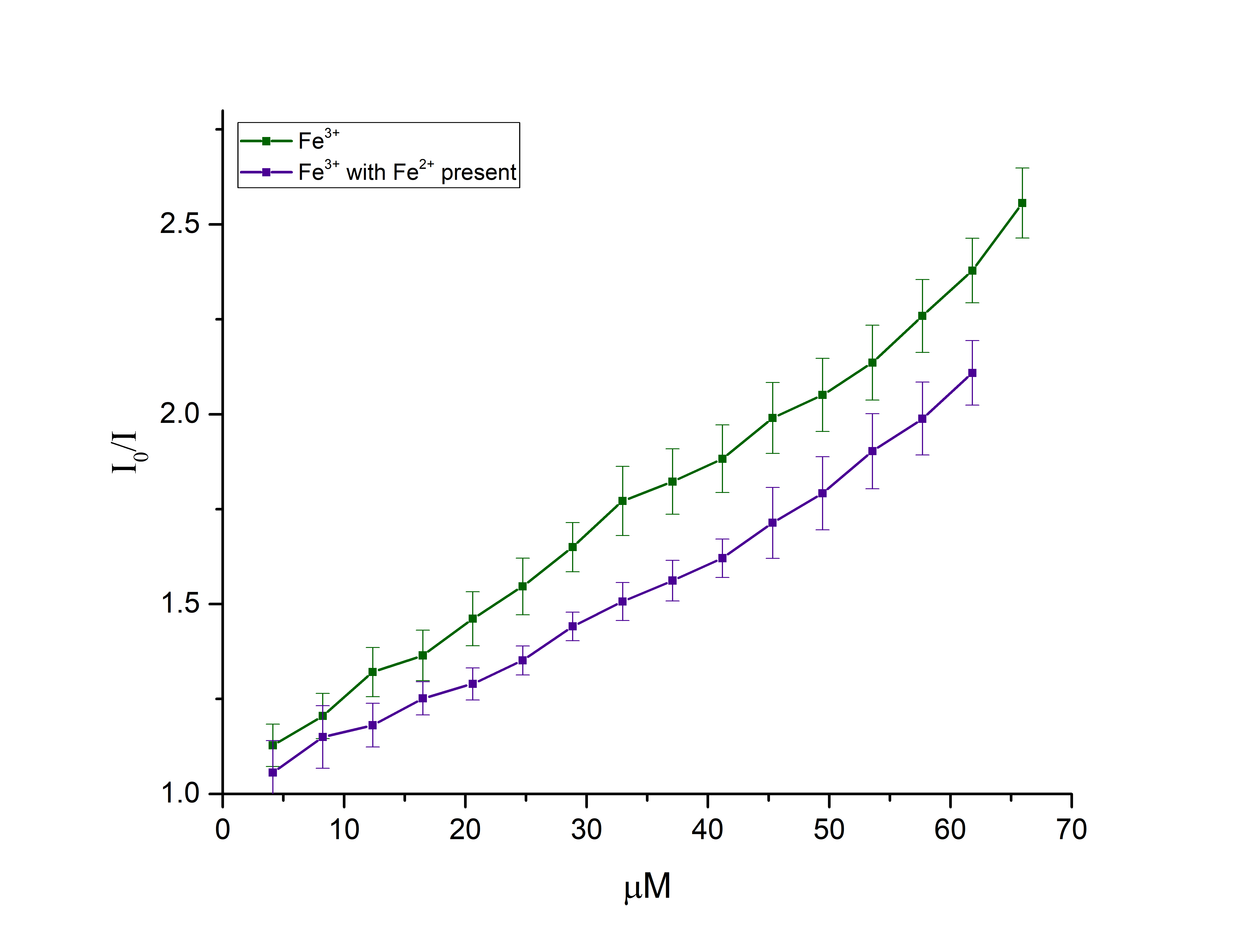


**Figure S22**. A relationship curve of the luminescence intensity and Fe^3+^ concentration with and without 32µM Fe^2+^ present. Error bars represent ± standard deviation (n = 3).


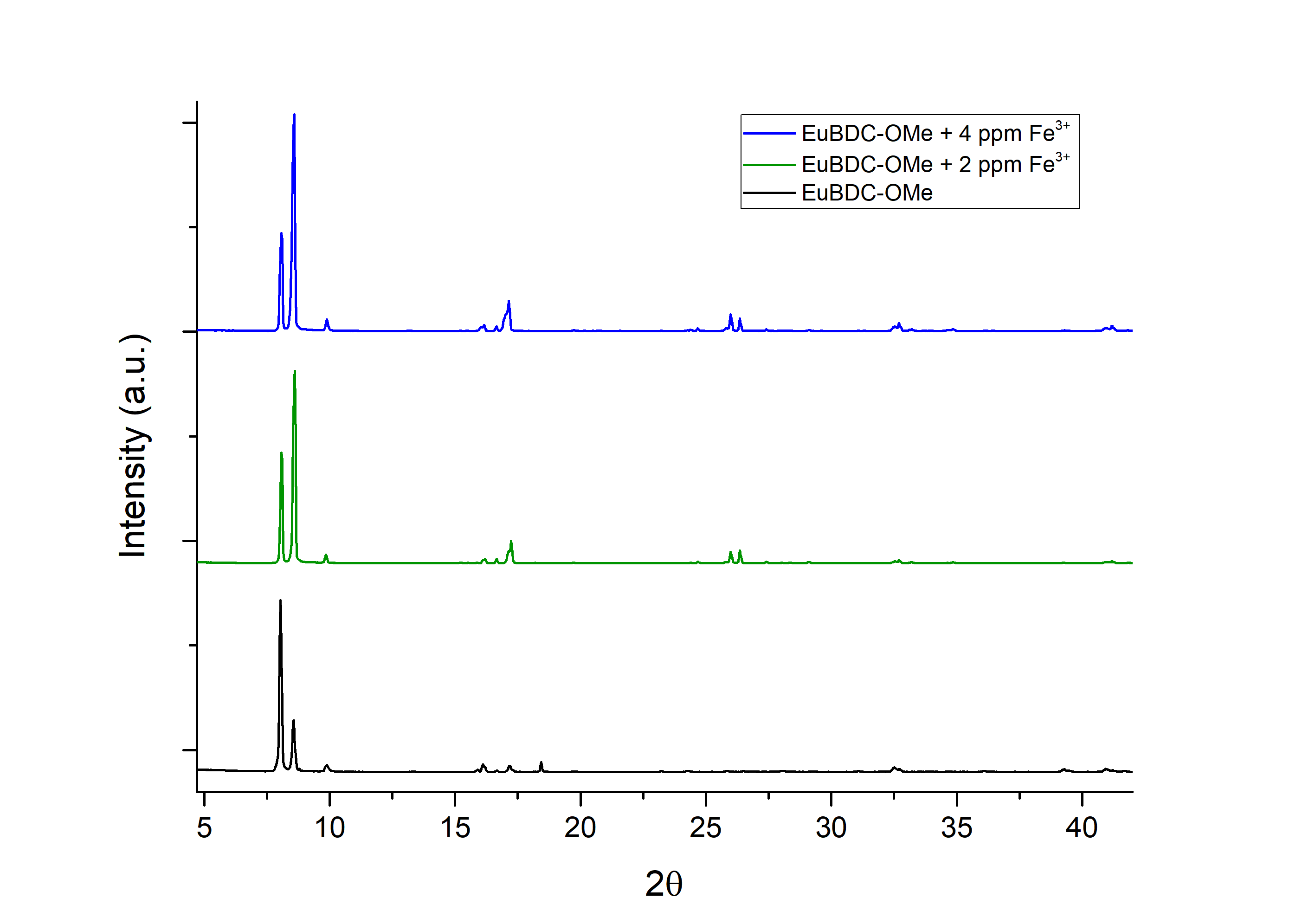


**Figure S23**. PXRD patterns of EuBDC-OMe soaked in 2 and 4 ppm Fe^3+^ containing solutions.


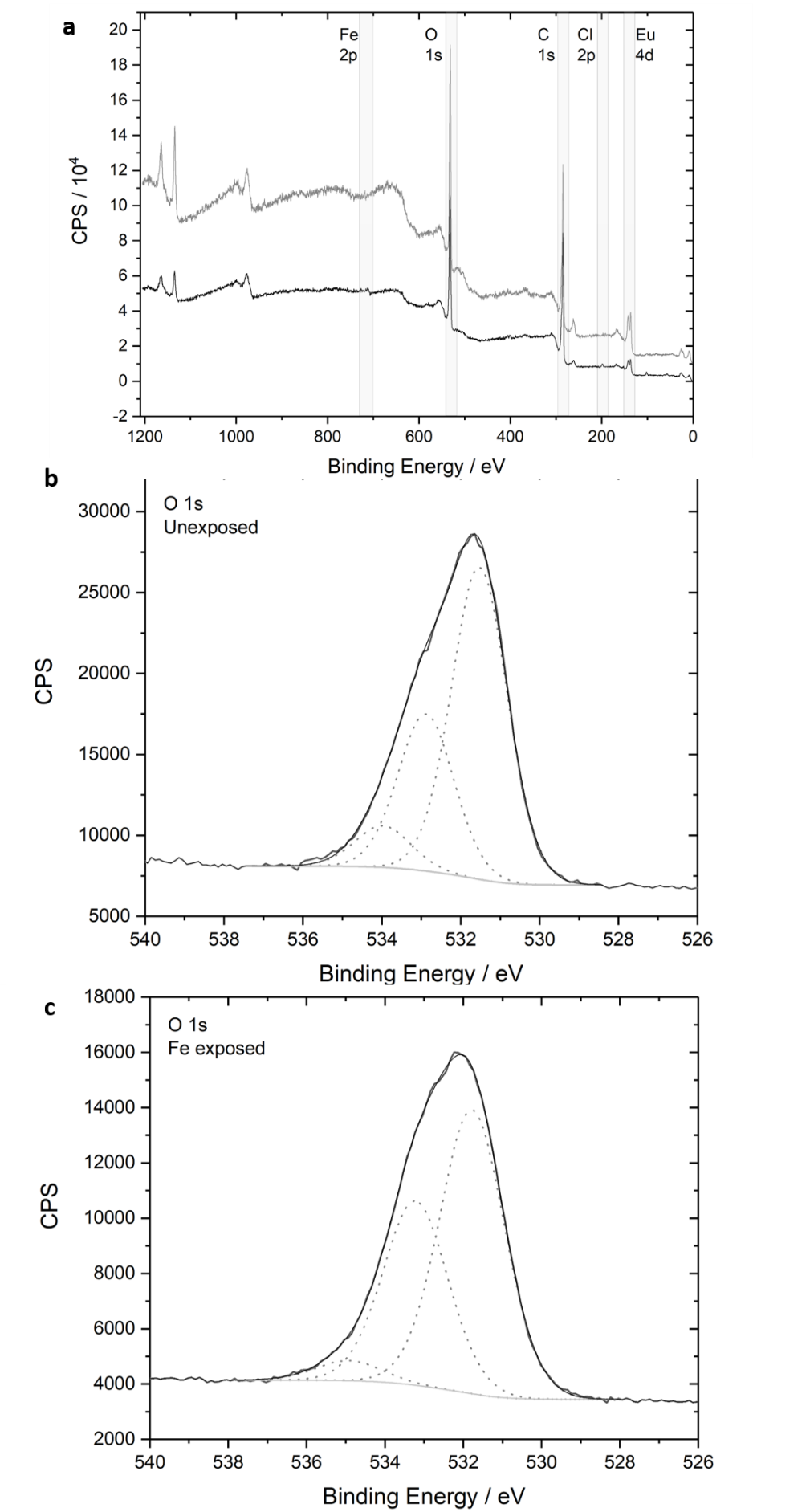


**Figure S24.** XPS spectra of EuBDC-OMe, with and without Fe^3+^ ions present: a) survey scan, showing the presence (and absence) of Fe atoms in the samples (Fe^3+^ treated sample at the bottom); b) high resolution (and fitted) O 1s peaks for untreated EuBDC-OMe; c) high resolution (and fitted) O 1s peaks for Fe^3+^ ion treated EuBDC-OMe.

**Table S4.** O 1s peak deconvolution, binding energies, abundances, widths, and assignments for of EuBDC-OMe, with and without Fe^3+^ present.

| **Element** | **Binding energy (eV)** | **Abundance^a^ (%)** | **FWHM (eV)** | **Assignment** |
| --- | --- | --- | --- | --- |
| **EuBDC-OMe O 1s** | 531.5 | 61.1 | 1.7 | C=O/ O–C=O bonds of carboxyl groups[24] |
|  | 532.9 | 30.8 | 1.7 | C–OH[24] |
|  | 534.0 | 8.2 | 1.7 | H_2_O |
| **EuBDC-OMe+ Fe^3+^ O 1s** | 531.8 | 58.1 | 2.0 | C=O/ O–C=O bonds of carboxyl groups[24] |
|  | 533.2 | 37.8 | 2.0 | C–OH[24] |
|  | 534.9 | 4.1 | 2.0 | H_2_O |


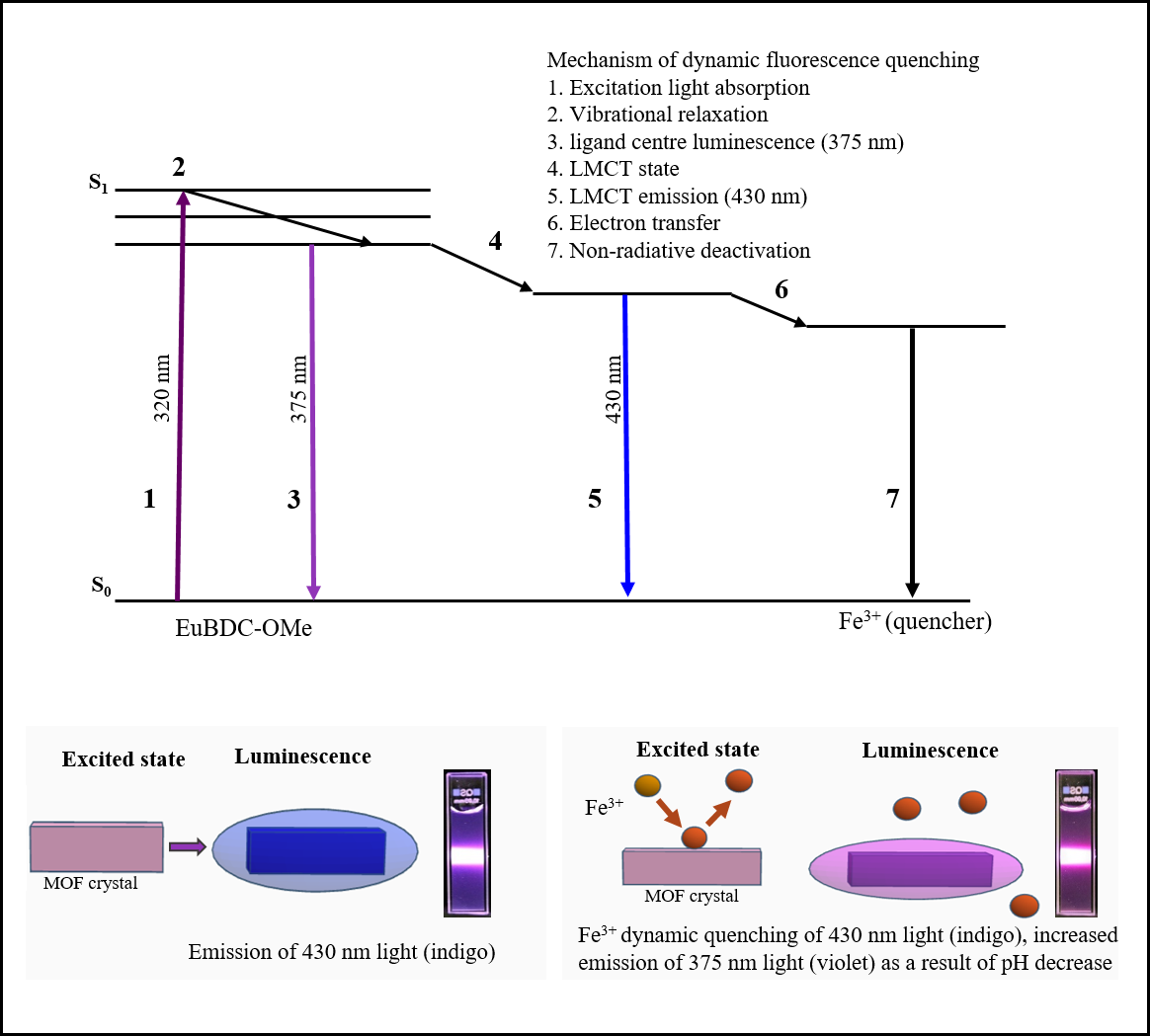


**Figure S25.** Energy transfer diagram demonstrating the Fe^3+^ dynamic quenching mechanism. Underneath is depicted the experiment for sensing, and the alteration of the color of the emission is shown in the included photographs of MOF suspensions illuminated by laser light at 320nm.

**References**

[1] H. Xu, Y. Dong, Y. Wu, W. Ren, T. Zhao, S. Wang, J. Gao, An -OH group functionalized MOF for ratiometric Fe3+ sensing, J. Solid State Chem. 258 (2018) 441–446. https://doi.org/10.1016/j.jssc.2017.11.013.

[2] Y. Zhang, B. Yan, A ratiometric fluorescent sensor with dual response of Fe3+/Cu2+ based on europium post-modified sulfone-metal-organic frameworks and its logical application, Talanta. 197 (2019) 291–298. https://doi.org/10.1016/j.talanta.2019.01.037.

[3] B. Ruan, J. Yang, Y.J. Zhang, N. Ma, D. Shi, T. Jiang, F.C. Tsai, UiO-66 derivate as a fluorescent probe for Fe3+ detection, Talanta. 218 (2020) 121207. https://doi.org/10.1016/j.talanta.2020.121207.

[4] H. Yu, Q. Liu, J. Li, Z.M. Su, X. Li, X. Wang, J. Sun, C. Zhou, X. Hu, A dual-emitting mixed-lanthanide MOF with high water-stability for ratiometric fluorescence sensing of Fe3+and ascorbic acid, J. Mater. Chem. C. 9 (2021) 562–568. https://doi.org/10.1039/d0tc04781c.

[5] P.-M. Chuang, J.-Y. Wu, A highly stable Zn coordination polymer exhibiting pH-dependent fluorescence and as a visually ratiometric and on–off fluorescent sensor, CrystEngComm. 23 (2021) 5226–5240. https://doi.org/10.1039/d1ce00705j.

[6] X. Guo, Q. Pan, X. Song, Q. Guo, S. Zhou, J. Qiu, G. Dong, Embedding carbon dots in Eu3+-doped metal-organic framework for label-free ratiometric fluorescence detection of Fe3+ ions, J. Am. Ceram. Soc. 104 (2021) 886–895. https://doi.org/10.1111/jace.17477.

[7] H. Xu, Y. Dong, Y. Wu, W. Ren, T. Zhao, S. Wang, J. Gao, An -OH group functionalized MOF for ratiometric Fe3+ sensing, J. Solid State Chem. 258 (2018) 441–446. https://doi.org/10.1016/j.jssc.2017.11.013.

[8] D. Aragão, J. Aishima, H. Cherukuvada, R. Clarken, M. Clift, N.P. Cowieson, D.J. Ericsson, C.L. Gee, S. Macedo, N. Mudie, S. Panjikar, J.R. Price, A. Riboldi-Tunnicliffe, R. Rostan, R. Williamson, T.T. Caradoc-Davies, MX2: a high-flux undulator microfocus beamline serving both the chemical and macromolecular crystallography communities at the Australian Synchrotron, J. Synchrotron Radiat. 25 (2018) 885–891. https://doi.org/10.1107/S1600577518003120.

[9] G.M. Sheldrick, No Title, Acta Crystallogr. Sect. A. 71 (2015) 3–8.

[10] G.M. Sheldrick, No Title, Acta Crystallogr. Sect. C. 71 (2015) 3–8.

[11] C.B. Hubschle, G.M. Sheldrick, B. Dittrich, ShelXle: a Qt graphical user interface for SHELXL, J. Appl. Crystallogr. 44 (2011) 1281–1284. https://doi.org/doi:10.1107/S0021889811043202.

[12] A. Thorn, B. Dittrich, G.M. Sheldrick, Enhanced rigid-bond restraints, Acta Crystallogr. Sect. A. 68 (2012) 448–451. https://doi.org/doi:10.1107/S0108767312014535.

[13] A. Spek, PLATON SQUEEZE: a tool for the calculation of the disordered solvent contribution to the calculated structure factors, Acta Crystallogr. Sect. C. 71 (2015) 9–18. https://doi.org/doi:10.1107/S2053229614024929.

[14] A. Spek, Structure validation in chemical crystallography, Acta Crystallogr. Sect. D. 65 (2009) 148–155. https://doi.org/doi:10.1107/S090744490804362X.

[15] D.E. Chandler, Z.K. Majumdar, G.J. Heiss, R.M. Clegg, Ruby crystal for demonstrating time- and frequency-domain methods of fluorescence lifetime measurements, J. Fluoresc. 16 (2006) 793–807. https://doi.org/10.1007/s10895-006-0123-7.

[16] J.R. Lakowicz, Time-Domain Lifetime Measurements, Princ. Fluoresc. Spectrosc. (1999) 95–140. https://doi.org/10.1007/978-1-4757-3061-6_4.

[17] Time‐resolved fluorescence lifetime measurements, HORIBA Sci. Time‐Resolved Fluoresc. Tech. Note TRFT‐1. (n.d.) 1–4.

[18] J.X.J. Zhang, K. Hoshino, Optical transducers: Optical molecular sensing and spectroscopy, 2019. https://doi.org/10.1016/b978-0-12-814862-4.00005-3.

[19] C.A.S. Tellez, E. Hollauer, M.A. Mondragon, V. Castano, Fourier transform infrared and Raman spectra, vibrational assignment and ab initio calculations of terephthalic acid and related compounds, Spectrochim. Acta Part A 57. 57 (2001) 993–1007.

[20] G. Wu, Y. Li, Y. Geng, X. Lu, Z. Jia, Adjustable pervaporation performance of Zr-MOF/poly(vinyl alcohol) mixed matrix membranes, J. Chem. Technol. Biotechnol. 94 (2019) 973–981. https://doi.org/10.1002/jctb.5846.

[21] E.D. Dikio, A.M. Farah, Synthesis, Characterization and Comparative Study of Copper and Zinc Metal Organic Frameworks, Chem. Sci. Trans. 2 (2013) 1386–1394. https://doi.org/10.7598/cst2013.520.

[22] J. Yang, A. Grzech, F.M. Mulder, T.J. Dingemans, Methoxy-modified MOF-5: A MOF-5 framework prepared by a mixed ligand approach, Eur. J. Inorg. Chem. (2013) 2336–2341. https://doi.org/10.1002/ejic.201201312.

[23] V.D. Doan, T.L. Do, T.M.T. Ho, V.T. Le, H.T. Nguyen, Utilization of waste plastic pet bottles to prepare copper-1,4-benzenedicarboxylate metal-organic framework for methylene blue removal, Sep. Sci. Technol. 55 (2020) 444–455. https://doi.org/10.1080/01496395.2019.1577266.

[24] S. Han, R.A. Ciufo, M.L. Meyerson, B.K. Keitz, C. Buddie Mullins, Solvent-free vacuum growth of oriented HKUST-1 thin films, J. Mater. Chem. A. 7 (2019) 19396–19406. https://doi.org/10.1039/c9ta05179a.
